# Anticholinergic Medication Burden Scales: A Systematic Review

**DOI:** 10.1111/jgs.70352

**Published:** 2026-02-23

**Authors:** Orla Vennard, Carrie Stewart, Mansi Tolia, Roy L. Soiza, Phyo K. Myint

**Affiliations:** ^1^ School of Medicine, Medical Sciences and Nutrition University of Aberdeen Aberdeen UK; ^2^ Ageing Clinical & Experimental Research Group, Institute of Applied Health Sciences University of Aberdeen Aberdeen UK; ^3^ Aberdeen Royal Infirmary, NHS Grampian, and Health Services Research Unit University of Aberdeen Aberdeen UK

**Keywords:** anticholinergic burden, older adults, polypharmacy, scales

## Abstract

**Background:**

Anticholinergic burden refers to the cumulative anticholinergic effect of all medications taken by an individual. Anticholinergic burden scales help identify patients at risk of anticholinergic adverse effects and guide prescribing. However, substantial variation exists between scales, with no gold standard identified. This variability may contribute to inconsistent risk assessment, suboptimal prescribing, and adverse outcomes.

**Aim:**

To systematically review available anticholinergic burden scales and their variability in medication lists, development strategies and scoring methods. As a secondary objective, the clinical outcomes associated with each scale were summarized.

**Methods:**

A systematic search was conducted up to January 2025. Studies proposing novel or updated anticholinergic burden scales were included. Two reviewers independently performed study selection, data extraction, and quality assessment, using a custom tool based on expert consensus and principles of scale development. Findings were narratively synthesized.

**Results:**

From 10,969 identified records, 21 studies met inclusion criteria. Medications included per scale ranged from 27 to 217, with 74% of high‐potency drugs scored inconsistently. Variability was influenced by geographical origin and methodology, with literature review followed by expert opinion the most common method of development. Dosage consideration, among others, was inconsistent across scales, affecting clinical relevance. Clinical outcome studies reflected such inconsistencies.

**Conclusion:**

No gold standard anticholinergic burden scale was identified. Scales with broader drug coverage and accounting for individual variability appeared more clinically relevant. This review highlights the need for a clinically accessible, universal scoring system to better address the risks associated with anticholinergic polypharmacy.

## Summary

- Key points
  ○ Substantial variety exists across anticholinergic burden scales in terms of included medication and assigned scores.
  ○ Differences in scale development methodologies and drug inclusion criteria contribute to uncertainty in anticholinergic assessments, with no gold standard or universally applicable scale currently available.
  ○ Efforts to standardize anticholinergic burden scales are needed to support consistent risk assessments and improve prescribing decisions for older adults.
- Why does this matter?
  ○ The use of anticholinergic medication is rising, disproportionately impacting older adults with multimorbidity and polypharmacy. This systematic review examines inconsistencies among anticholinergic burden scales, which may contribute to inadequate medication reviews and suboptimal prescribing in geriatric care.

## 1 Introduction

Anticholinergic (ACH) medications have contributed significantly to rising polypharmacy, with prescriptions increasing nine‐fold between 1990 and 2015 [1]. Anticholinergic activity, resulting from inhibition of acetylcholine, is therapeutically desirable in drugs such as oxybutynin, but can occur unintentionally with medications like methyldopa, leading to adverse effects (Figure S1).

Anticholinergic activity is associated with a range of adverse effects including confusion, cognitive impairment, and parasympathetic symptoms such as dry mouth and constipation [2, 3]. Prolonged use is linked to an increased risk of falls, hospital admissions, and General Practitioner visits [4]. After three years of ACH exposure, individuals have an estimated 50% greater risk of developing dementia [5]. The cumulative exposure, termed anticholinergic burden (AChB) [6], is associated with a 26% increase in mortality risk for each unit increase in AChB [7].

Despite recommendations to minimize ACH prescribing, around 50% of older adults are prescribed at least one ACH medication [8]. ACH prescribing is strongly associated with multimorbidity, disproportionately affecting older adults, females, and those of lower socioeconomic status [1].

Older adults are particularly susceptible to AChB due to age‐related pharmacokinetic and pharmacodynamic changes—such as reduced acetylcholine reserve, renal excretion, and hepatic clearance, consequently increasing drug half‐life and subsequent adverse effects [9, 10]. Quetiapine, with high anticholinergic activity, is frequently prescribed in these groups; notably, its clearance decreases by up to 50% with advancing age [9]. Additionally, AChB‐related effects often mimic aging symptoms, complicating clinical assessment.

Scoring systems have been developed to identify patients at risk of ACH‐related adverse effects. However, their clinical utility remains limited—only 37% of British clinicians can accurately assess AChB [11]. Significant variation exists between scales in drug inclusion and scores assigned; for example, quetiapine is rated as low, moderate, or high depending on the scale [12]. These discrepancies complicate clinical decision‐making and may increase inappropriate prescribing and adverse events.

AChB scales are developed using diverse methodologies, including in vitro radioreceptor assays, serum anticholinergic activity (SAA) assays, literature‐based medication rankings, and expert consensus, each with varying levels of evidence and clinical applicability (Figure S2).

Previous reviews have examined factors contributing to the heterogeneity among AChB scales, including differences in methodological approaches such as average daily dose versus cumulative dose, and the subsequent inter‐scale correlation [13, 14, 15, 16]. However, these reviews are limited in scope, covering only scales published before October 2023 and excluding scales employing laboratory‐based methodologies, equation‐derived scoring systems [14, 15], or approaches combining potency and dosage in cumulative burden calculation [13]. Other reviews have focused on the clinical implications of AChB, including the predictive validity of scales in specific populations [12, 14, 17, 18, 19, 20, 21], and their integration into clinical practice [2, 22]. Despite these contributions, a comprehensive up‐to‐date evaluation of the methodological approaches used in AChB scale development and their contribution to variability between scales is lacking.

To address these gaps, this review provides an updated comprehensive evaluation of AChB scales, guided by the central question: What heterogeneity currently exists between AChB scales and what are the factors underlying this variability? Three key components of the scale design and development were systematically analyzed: (i) the range of methodologies used in the development of each scale, (ii) the medications included in each scale, (iii) the medications that receive a high score across the scales identified. As a secondary objective, a citation analysis was conducted to identify and summarize the clinical outcomes associated with each scale. This approach enables an assessment of current progress toward establishing the clinical utility of AChB scales in the context of methodological variability.

## 2 Methods

A systematic review of studies and relevant literature was conducted to identify published novel or updated anticholinergic burden scales. The review follows PRISMA guidelines (Table S1) and was prospectively registered on PROSPERO (ID: CRD42017076510).

### 2.1 Search Strategy

The following databases were searched from inception to January 2025: Ovid MEDLINE, Ovid MEDLINE Epub Ahead of Print, Ovid MEDLINE In‐Process & Other Non‐Indexed Citations, Ovid EMBASE and OVID PsycINFO. Search terms included those related to anticholinergics (e.g., *anticholinergic, anti‐cholinergic, cholinergic antagonist, antimuscarinic, anti‐muscarinic or muscarinic antagonist*) and to scales (e.g., *scale*, *score*, *rank*, *rating*, *grading*, *index*, *classification*).

The search strategy was validated for Ovid MEDLINE and adapted for other databases. Reference lists of included papers and previously published AChB systematic reviews were manually screened to identify additional relevant studies. The full search strategies for each database are provided in Tables S2–S4.

### 2.2 Eligibility

To be eligible for this systematic review studies were required to meet the following criteria: (i) provides a finite list of anticholinergic medications, (ii) assigns ordinal scores based on anticholinergic potency or risk, (iii) proposes a novel or updated scale, (iv) describes the methodology used in scale development, (v) study participants, where applicable, are human, (vi) studies published in any language and conducted in any setting. The following exclusion criteria were applied: (i) conference abstracts, (ii) dissertations, (iii) case studies letters.

## 3 Data Collection and Analysis

### 3.1 Study Selection

Search results were imported into ProQuest RefWorks and duplicates were removed. Four independent reviewers (Authors O.V., C.S., P.K.M. and R.L.S.) screened titles and abstracts against the inclusion and exclusion criteria. Full texts of potentially eligible studies were obtained and reviewed independently. Where full texts could not be found, study authors were contacted.

### 3.2 Data Extraction and Synthesis

Data were extracted from each included study by four independent reviewers (O.V., C.S., P.K.M., and R.L.S.) using a pre‐planned and piloted extraction form (Table S5). Study authors were contacted where clarification was required, and discrepancies were resolved through discussion between authors.

Data were summarized descriptively to depict the year of publication; country of origin; number of ACH medications listed; scoring system used; and an overview of the methodology used to compose the scale.

Medications were ranked by their frequency across scales and assigned scores using a standardized four‐point system (0–3). For the AAS [23], scores of 2 and 3 were combined and assigned a value of 2 to align with the four‐point format used in most other scales. In Chew et al. [24] scale, two columns representing 0 and 0/minimal anticholinergic activity were combined into a single score of 0. Two [25, 26]‐ and three‐point scales [27, 28, 29, 30, 31, 32] were incorporated directly, with the scores in Table 2, matching those reported in the original scales. All data were collated in an Excel spreadsheet.

To address the secondary objective, a citation analysis of the original scale development papers was conducted to identify the studies that evaluated the clinical outcomes associated with each.

### 3.3 Quality Assessment

Quality assessment was undertaken by independent reviewers (O.V., C.S., P.K.M. and R.L.S.), using a custom tool informed by expert knowledge of the researchers and current understanding of scale development (Figure S3). Appropriate methods for reaching expert consensus were considered as methods that do not introduce bias into the medication rankings. Scales were ranked according to the robustness of their development process.

## 4 Results

**FIGURE 1:**
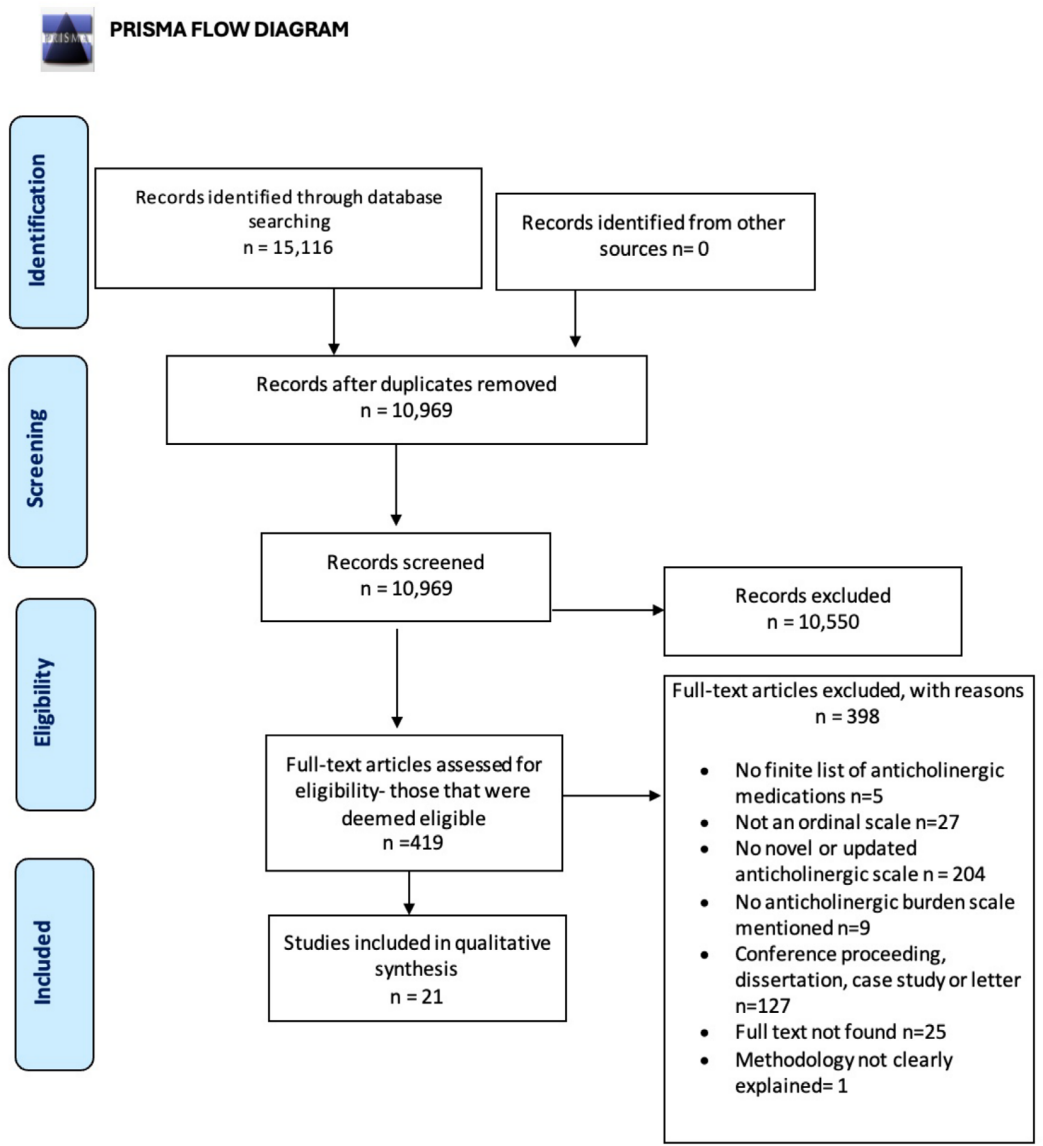
Prisma flow diagram illustrating the study selection process.

After deduplication, the title and abstract of 10,969 papers were screened for potential eligibility (Figure 1). A total of 419 papers were retrieved for full‐text review, of which 398 were excluded based on the eligibility criteria.

### 4.1 Scale Characteristics

The characteristics of the 21 included scales and their development are presented in Table 1, with additional details of scale development provided in Table S5.

**TABLE 1:** Overview of AChB scales, including their methodological development and country of origin.

AChB medication scale	Country of origin^a^	Number of ACH medications listed^b^	Scoring system	Basis of methodology
Summers list [20]	USA	67	Scale from 1 to 3	Existing literature
ABC [16]	France	27	Scale from 0 to 3	Existing literature and expert opinion
ADS [21]	USA	117	Scale from 0 to 3	Existing literature: Serum drawn from subjects. Radioreceptor assay.
ARS [22]	USA	49	Scale from 0 to 3	Extensive literature review and expert opinion
ACB [23]	USA	88	Scale from 0 to 3	Existing literature and expert opinion
Chews list [14]	USA	39	Scale of 0, 0/+, +, ++ or +++	SAA data
Ehrt et al. [23]	Norway	29	Scale from 0 to 4	Based on list by Chew et al. and existing literature
ACL [28]	Australia	49	Scale from 0 to 3	Existing literature and expert opinion
Ellett's list [18]	Australia	31^b^	Score of 2 or 3	Existing literature
mARS [29]	UK	61	Scale from 1 to 3	Based on ARS by Rudolph et al., existing literature and expert opinion
AEC [30]	UK	60	Scale from 0 to 3	Existing literature
AIS [27]	France	128	Scale from 1 to 3	Existing literature and expert opinion.
Marante [19]	Belgium	49^b^	Score of 1 or 2	Existing literature, expert opinion and daily dosage calculations
ABS [31]	Germany	153	Score from 0 to 3	Existing literature and expert opinion
KABS [32]	Korea	138	Score from 0 to 3	Existing literature and expert opinion
BAAS [24]	Brazil^a^	125	Score from 1 to 3	Existing literature
CALS [17]	Spain	217	Score from 1 to 3	Existing literature and expert opinion
SweABS [26]	Sweden^a^	104	Score from 0 to 3	Existing literature and expert opinion
Kehman [33]	Denmark	87	Score from 1 to 3	Existing literature
ABS [25]	Japan^a^	96	Score from 0 to 3	Muscarinic receptor‐binding activities
JARS [34]	Japan	158	Score from 1 to 3	Existing literature and expert opinion

*Note*: Scales listed in chronological order by year of development, with the most recent at the bottom.

Abbreviations: ABC, anticholinergic burden classification; ACB, anticholinergic cognitive burden; ACH, anticholinergic; AChB, anticholinergic burden; ACL, anticholinergic load scale; ADS, anticholinergic drug scale; AEC, anticholinergic effect on cognition; AIS, anticholinergic impregnation scale; ARS, anticholinergic risk scale; BAAS, brazilian anticholinergic activity drug scale; CALS, CRIDECO anticholinergic loading scale; JARS, Japanese anticholinergic risk scale; KABS, Korean anticholinergic burden scale; MARANTE, Muscarinic acetylcholinergic receptor antagonist exposure scale; mARS, modified anticholinergic risk scale; SAA, serum anticholinergic activity; SweABS, Swedish anticholinergic burden scale.

^a^ Indicates the country from which the list of medications included in the scale originates, e.g., the country in which patient drug charts or prescribing records were analyzed.

^b^ Excluding medications determined to have no anticholinergic activity (score = 0) where applicable.

The number of medications included in each scale ranged from 27 [35] to 217 [32]. Ten scales used a four‐point scoring system from 0 to 3 to rank medication potency. Others ranged between two‐ and five‐point scales. Some scales excluded medication with no clinically relevant anticholinergic activity [25, 26], whereas Chews list [24], featured only 22.7% of medication with significant ACH effects.

The 21 scales arise from different countries, with five developed in the USA [24, 27, 33, 34, 36]. Scales that alter their medication list according to drugs registered in the country of development include the BAAS [37], ABS [38] and Swe‐ABS [39].

### 4.2 Rankings of Medication Across Scoring Systems

The 25 medications that were most frequently included are summarized in Table 2. Medications received a score of low, moderate, or high ACH potency across the 21 scales.

**TABLE 2:** Top 25 anticholinergic medications ranked by cumulative score across included scales.

No.	Medication	ATC drug classification	Main indication	Scoring frequency	Score 3	Score 2	Score 1	Score 0
1	Amitriptyline	N06A	Neuropathic pain	21	20	1		
2	Atropine	A03B	Bradycardia	20	20			
3	Oxybutynin	G04B	Urinary frequency/incontinence	18	16	2		
4	Clomipramine	N06A	Depression	16	15	1		
5	Hydroxyzine	N05B	Pruritus	16	14	1	1	
6	Benzhexol/trihexyphenidyl	N04A	Parkinson's disease	15	15			
7	Olanzapine	N05A	Schizophrenia	17	6	10	1	
8	Diphenhydramine	R06A	Allergy	13	12	1		
9	Cyproheptadine	R06A	Allergy	13	10	3		
10	Clozapine	N05A	Psychosis	12	11	1		
11	Promethazine	R06A	Allergy	13	11		2	
12	Trimipramine	N06A	Depression	12	11	1		
13	Doxepin	N06A	Depression	12	11	1		
14	Tolterodine	G04B	Urinary frequency/incontinence	12	10	2		
15	Nortriptyline	N06A	Depression	12	9	3		
16	Levomepromazine	N05A	Psychosis	14	5	9		
17	Paroxetine	N06A	Depression	16	3	11	2	
18	Hyoscine/Scopolamine	A04A	Motion sickness	11	11			
19	Chlorphenamine	R06A	Allergy	11	10	1		
20	Thioridazine	N05A	Psychosis	10	10			
21	Benztropine	N05A	Parkinson's disease	10	10			
22	Orphenadrine	N04A	Parkinson's disease	10	10			
23	Imipramine	N04A	Depression	9	9			
24	Tizanidine	M03B	Muscle relaxant	9	9			
25	Chlorpromazine	N05A	Psychosis	9	8	1		

*Note*: Medications are ordered by total cumulative score across 21 included AChB scales, reflecting both frequency of inclusion and assigned potency. 0 = no anticholinergic effect, 1 = minimal anticholinergic effect, 2 = moderate anticholinergic effect, 3 = strong anticholinergic effect. A03B = belladonna and derivatives, A04A = antiemetics and anti‐nauseants, G04B = urologicals, M03B = muscle relaxants, N04A = anticholinergic agents, N05A = psycholeptics, N05B = anxiolytics, N06A = antidepressants, R06A = Antihistamines for systemic use. Green: No anticholinergic activity. Orange: Moderate anticholinergic activity Pink: Strong anticholinergic activity. Yellow: Weak anticholinergic activity.

Abbreviation: ATC, anatomical therapeutic chemical.

### 4.3 Quality Assessment of AChB Scale Development Process

Methods used in scale development included: in vitro radioreceptor assay (*n* = 2) [24, 38], SAA measurement (*n* = 2) [29, 34], composite methodology of expert and literature opinion (*n* = 14) [23, 26, 28, 29, 30, 31, 33, 35, 36, 39, 40, 41, 42, 43], or literature reviews only (*n* = 4) [25, 27, 30, 37] (Table 1, Table S6). Appropriate methods to resolve disagreements were reported for eight scales, with one scale [31] excluding medication in which an agreement could not be reached. The most common development method used in 11 of the included scales combines literature reviews with expert opinion [26, 28, 29, 32, 33, 35, 36, 39, 40, 41, 42]. All 13 of the highest‐rating scales for development robustness [23, 26, 28, 29, 30, 33, 35, 36, 39, 40, 41, 42, 43] (Table 3) involve expert panels of at least two members, with eight [26, 31, 35, 36, 39, 40, 43] incorporating multidisciplinary input.

**TABLE 3:** Quality assessment of the 21 included anticholinergic burden (AChB) scales.

ACB scales	ABS [25]	Ellett's list [18]	Kehman et al. [30]	BAAS [24]	Summers' list [20]	mARS [29]	Chew's list [14]	ADS [21]	Ehrt et al. [15]	CALS [17]
1. Is the methodology clearly described?	Yes	Yes	Yes	Yes	Yes	Yes	Yes	Yes	Yes	Yes
2. Is the scale based on composite methodology? (e.g., combination of expert opinion, existing literature, in vitro data etc.)	No	No	Yes	No	No	Yes	No	Yes	Yes	Yes
a. If literature based, was an appropriate systematic literature search described?	N/A	No	Yes	Yes	No	No	No	No	No	Yes
3. Does the scale include expert opinion?	No	N/A	No	Yes	No	Yes	No	No	Yes	Yes
a. How many experts? (=3 = good, 2 = fair, 1 = poor)	N/A	N/A	N/A	NR	N/A	NR	N/A	N/A	Fair (2)	Good (3)
b. Were the experts multidisciplinary?	N/A	N/A	N/A	NR	N/A	NR	N/A	N/A	No	No
c. What method was used for experts to reach a consensus (e.g., Delphi method etc.)	N/A	N/A	N/A	NR	N/A	NR	N/A	N/A	NR	Expert panel
d. Is the method for resolving disagreements between experts reported and appropriate?	N/A	N/A	N/A	NR	N/A	NR	N/A	N/A	NR	NR
4. Does the scale take into account the medication dose?	No	No	No	No	Yes	No	Yes	Yes	No	No
5. Does the scale take into account the duration of medication use?	No	No	No	No	No	No	No	Yes	No	No
6. Does the scale consider all routes of medication administration?	No	No	No	No	No	Yes	NR	Yes	Yes	No
7. Was the scale designed with specific outcome(s) in mind?	No	No	No	No	Yes	No	Yes	No	Yes	No
8. Does the scale subsequently show positive association with the intended specific outcome(s) (i.e., has been validated against the clinical outcome)?	No	Yes	No	Yes	Yes	Yes	Yes	Yes	Yes	Yes

ACB scales	JARS [34]	Swe‐ABS [26]	ABS [31]	ABC [16]	ACB [23]	KABS [32]	AIS [27]	ARS [22]	ACL [28]	AEC [30]	MARANTE [19]
1. Is the methodology clearly described?	Yes	Yes	Yes	Yes	Yes	Yes	Yes	Yes	Yes	Yes	Yes
2. Is the scale based on composite methodology? (e.g., combination of expert opinion, existing literature, in vitro data etc.)	Yes	Yes	Yes	Yes	Yes	Yes	Yes	Yes	Yes	Yes	Yes
a. If literature based, was an appropriate systematic literature search described?	Yes	Yes	Yes	No	Yes	Yes	No	Yes	No	Yes	No
3. Does the scale include expert opinion?	Yes	Yes	Yes	Yes	Yes	Yes	Yes	Yes	Yes	Yes	Yes
a. How many experts? (=3 = good, 2 = fair, 1 = poor)	Good (6)	Good (4)	Good (3)	Good (3)	Good (6)	Good (7)	Good (5)	Good (3)	Good (4)	Fair (2)	Good (6)
b. Were the experts multidisciplinary?	Yes	Yes	No	Yes	Yes	Yes	No	No	Yes	Yes	Yes
c. What method was used for experts to reach a consensus (e.g., Delphi method etc.)	Delphi method	Independ‐ent reviewer	Expert Panel	NR	NR	Delphi method	Statistical method	Statistical Method	Statistical method	Independ‐ent Reviewer	Expert panel
d. Is the method for resolving disagreements between experts reported and appropriate?	No	Yes	Yes	NR	N/A	Yes	Yes	Yes	Yes	Yes	Yes
4. Does the scale take into account the medication dose?	No	No	No	No	No	No	No	No	No	No	Yes
5. Does the scale take into account the duration of medication use?	No	No	No	No	No	No	No	NR	No	No	No
6. Does the scale consider all routes of medication administration?	No	No	No	Yes	NR	No	Yes	No	Yes	Yes	Yes
7. Was the scale designed with specific outcome(s) in mind?	No	No	No	Yes	Yes	No	Yes	Yes	Yes	Yes	Yes
8. Does the scale subsequently show positive association with the intended specific outcome(s) (i.e., has been validated against the clinical outcome)?	No	No	Yes	Yes	Yes	Yes	Yes	Yes	Yes	Yes	Yes

*Note*: Summary of the methodological approaches used in the development of each scale, and factors considered when assigning medication scores. Scales are arranged from left to right, in order of increasing methodological robustness. Green: Increased robustness of scale development process. Red: Decreased robustness of scale development process. Yellow: Not applicable/Not reported.

Abbreviations: ABC, anticholinergic burden classification; ACB, anticholinergic cognitive burden; ACL, anticholinergic load scale; ADS, anticholinergic drug scale; AEC, anticholinergic effect on cognition; AIS, anticholinergic impregnation scale; ARS, anticholinergic risk scale; BAAS, Brazilian anticholinergic activity drug scale; CALS, CRIDECO anticholinergic loading scale; JARS, Japanese anticholinergic risk scale; KABS, Korean anticholinergic burden scale; MARANTE, Muscarinic acetylcholinergic receptor antagonist exposure scale; mARS, modified anticholinergic risk scale; N/A, not applicable; NR, not reported; SweABS, Swedish anticholinergic burden scale.

Four scales considered medication dose during scale development [24, 26, 27, 34] (Table 3). Dosages were primarily considered by creating dose ranges for the included medication and adjusting the AChB score accordingly [26, 27, 34]. This included maximum recommended dose [34], therapeutic and sub‐therapeutic drug levels [27], and minimal, maximal effective and maintenance dose [26]. Chew et al. [24] created estimated dose‐anticholinergic‐activity curves and drugs were categorized according to their maximum anticholinergic activity. Ten scales excluded routes with little systemic absorption [25, 27, 28, 33, 37, 39, 40, 41, 42, 44]. Other factors considered included BBB penetration of drugs [35, 36, 43] and drug–drug interactions [35].

### 4.4 Clinical Applicability and Clinical Outcome Studies

Three scales offer practical guidance for interpreting the AChB score in clinical settings [28, 36, 43]. Bishara et al. recommend avoiding medications scored one to three and advise discontinuing those scored two or three in cognitively impaired patients. Others [28, 45] suggest medication alternatives or dose adaptations.

**FIGURE 2:**
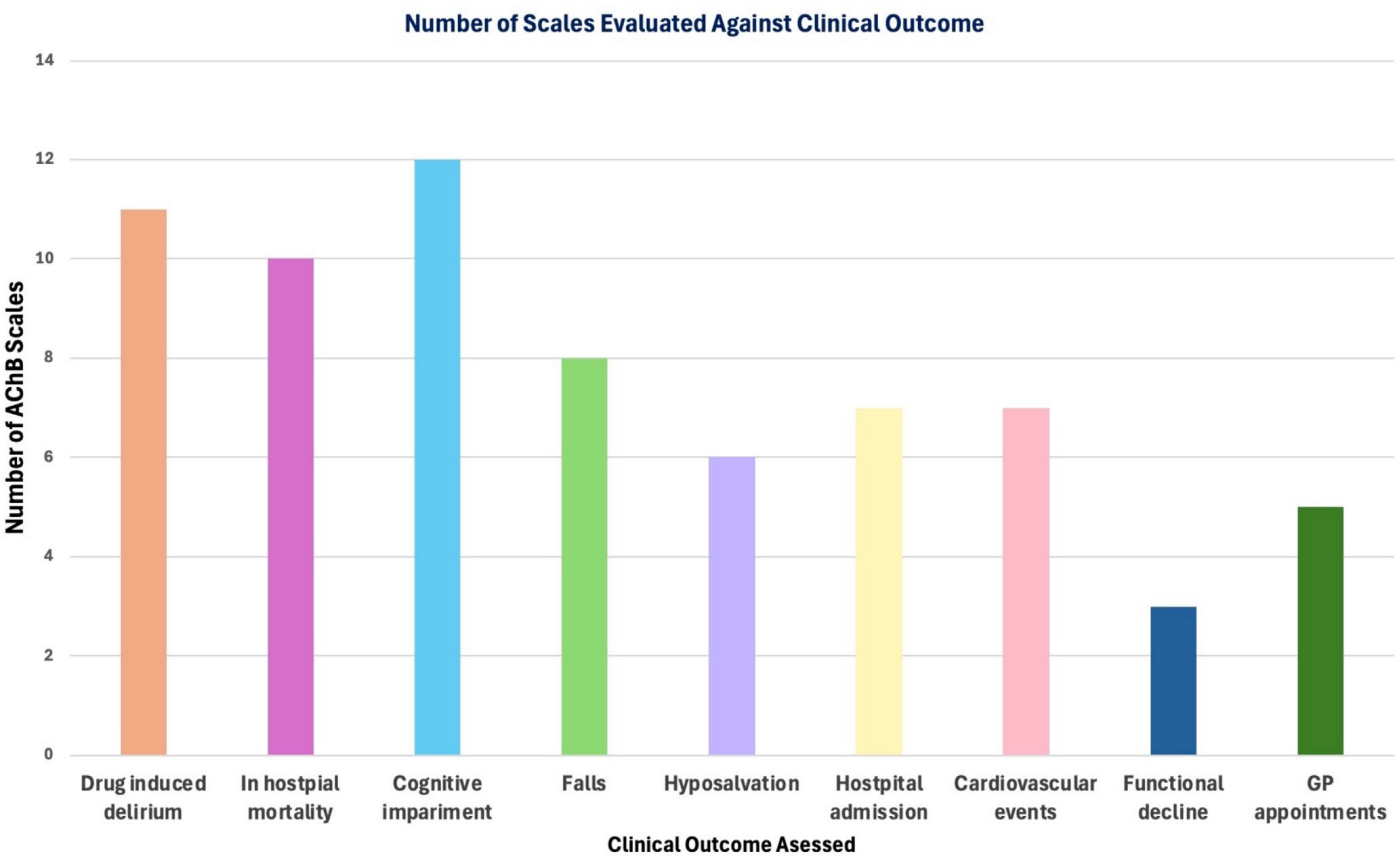
Evaluation of AChB scales against clinical outcomes. Number of anticholinergic burden (AChB) scales evaluated against specific clinical outcomes, identified through citation analysis of the included studies. The *X*‐axis shows the clinical outcome assessed, whereas the *Y*‐axis indicates the number of AChB scales evaluated against each outcome. Refer to Table S6 for details of clinical outcome studies. AChB, anticholinergic burden; GP, general practitioner.

Eight AChB scales were evaluated for associations with clinical outcomes within their development papers [23, 25, 27, 29, 32, 33, 35, 40]. In addition, nine other scales [24, 28, 29, 34, 36, 37, 41, 42, 43], have been assessed in external studies, examining relationships between AChB scores and clinical outcomes (Figure 2). Nine common outcomes included in such studies are: drug‐induced delirium, in‐hospital mortality/all‐cause mortality, cognitive impairment, falls, hyposalivation, hospital admissions, cardiovascular events, functional decline, and general practitioner appointments, with most studies focusing on older adults. Clinical outcome studies included in Figure 2 (further details in Table S7) showed a positive correlation between the AChB scale and the outcome investigated, whereas three studies [44, 46, 47] found limited correlation. The four most recent scales [30, 31, 38, 39] lack evaluation against a clinical outcome due to their recent publication; however, they were included to ensure a comprehensive review.

## 5 Discussion

This systematic review provides a comprehensive, up‐to‐date evaluation of AChB scales, examining their methodological development and the factors contributing to heterogeneity. Twenty‐one scales were identified, including those not evaluated in previous reviews [30, 31]. Substantial variation exists in both medication inclusion and scoring, even among the more recently developed scales [30, 31, 38, 39] highlighting the continued challenge of creating a universally applicable and methodologically robust tool.

No universal consensus exists on the most appropriate AChB scale. The number of medications included varies widely: for example, Ancelin et al. [35] selected medications from a cohort from the south of France, whilst Ramos et al. [32] based the medication listings on previous scales (Table 1). Broader drug coverage may better reflect diverse prescribing practices, proving a more universally applicable tool. Other factors influencing drug inclusion include development location, date and ACH potency. Regional differences limit applicability, such as BAAS [37], ABS [38] and the Swe‐ABS [39], which only include medications licensed in their country of origin (Table 1). Variations in drug availability, healthcare systems, and prescribing cultures [48]—even within Europe [49]—mean that AChB scales, which mainly originate from high‐income countries (Table 1), may not reflect AChB in lower‐income settings, where the adverse effects of polypharmacy are just as prevalent [50].

The range of medication included in the scale can depend on ACH potency. Some scales, such as Kalisch Ellett et al. [25] and Klamer et al. [26] included only higher scoring medication, which were likely to impact clinical outcomes (Table 1). Low‐potency drugs, particularly at low doses, may have minimal clinical impact, with drugs scoring 2 or 3 correlating greatest to increased falls risk [51]. Nevertheless, low‐potency drugs can contribute substantially to the overall AChB [37], and may improve scale transparency when included [43]. Further research is needed to compare scales including both low‐ and high‐potency anticholinergic activity drugs versus only high‐anticholinergic activity drugs.

Moreover, scales, such as the ACB [35] and ADS [34], risk being outdated, omitting newer drugs, such as fesoterodine [52] and potentially underestimating AChB [32]. Newer scales with broader drug coverage can improve applicability across a diverse range of clinical settings [28, 29, 39, 40, 41, 42]. These findings highlight the value of developing internationally applicable, routinely updated scales that enhance the relevance of AChB assessments globally.

Even where scales agree on the included medication, rankings often differ –74% of top 25 highest‐scoring drugs receive inconsistent scores (Table 2). Some drugs, such as atropine and amitriptyline, consistently score 3 across all scales, highlighting their ACH risk and supporting appropriate prescribing [53]. In contrast, olanzapine and paroxetine are rated as low, moderate or high depending on the scale used (Table 2), potentially compromising risk assessment and scale reliability. These inconsistencies [10, 13] remain evident across more recently developed scales, underscoring the ongoing need for methodological standardization.

Scoring systems employ various approaches to assign final rankings. Expert‐based methods can introduce bias if not standardized. For example, JARS [31] excluded medications when consensus could not be reached, whereas scales developed by fewer or single‐discipline teams are more prone to bias. Nevertheless, expert input enhances scales' clinical relevance, improving their ability to predict the impact of ACH medications on patient outcomes [15, 26, 35, 54]. A diverse multidisciplinary team, such as that used in KABS, can strengthen scale development and clinical applicability [42]. Expert input may also improve alignment with clinical outcomes and biological markers of anticholinergic activity [14, 54], supporting more accurate predictions than literature‐ or database‐based methods.

Notably, scales without a composite methodology (Table 3) rank among the lowest five for robust development. This aligns with existing literature, suggesting that mixed‐method approaches—combining literature evidence, pharmacological data, and expert input—are most effective by aligning biological precision with clinical outcomes [45], as seen in more recently developed scales [30, 31, 32, 39] (Table 3). The lack of methodological standardization likely contributes to inconsistent scoring, undermining the reproducibility and clinical confidence in these tools.

Inconsistent rankings are further influenced by differing clinical factors and inter‐individual variations considered during scale development. Key factors including dose, administration route, and blood–brain barrier (BBB) permeability are not consistently integrated into scales, factors particularly relevant to older adults, those most vulnerable to ACH effects [9, 13, 37]. Low‐potency ACH drugs can cause significant adverse effects at high doses for extended durations [31]. However, only four scales—developed before 2017—account for dosage, and methods for dose adjustment remain non‐standardized [24, 26, 27, 34].

The MARANTE scale represents methodological advancement, incorporating both potency and dosage [26], receiving the highest score for development robustness (Table 3). However, its complexity and omission of factors such as BBB permeability and renal function limit its practical use to population‐level assessment rather than individual patient assessment. Even among scales accounting for drug dose and potency, the methodology varies and factors including adherence and ACH sensitivity are often overlooked [22]. To enhance clinical utility, the ability to predict and guide clinically meaningful prescribing decisions, future scale development should integrate these key variables of anticholinergic exposure and response [14, 55].

While this review did not systematically assess clinical implementation or deprescribing outcomes, evidence of clinical evaluation was summarized through a secondary citation analysis. A clearer understanding of how scoring systems are developed and associated with clinical outcomes can support more informed selection of tools in medication reviews and deprescribing decisions.

Most scales have been associated with at least one clinical outcome (Table 3)‐ most commonly cognitive impairment, with others including peripheral ACH effects [26, 33] and hospitalization due to confusion dementia [25]. Studies predominantly focus on older adults: the demographic most vulnerable to ACH adverse effects [1]. Most clinical outcome studies are observational due to ethical constraints (Table S3); however, they consistently show a positive correlation between increased AChB scores and adverse clinical outcomes [56]. Kersten et al. [57] conducted a randomized control trial assessing whether reducing AChB score improves cognitive outcomes in older adults, demonstrating the efforts to establish a causal relationship between an increased AChB and clinical outcomes.

Evaluating how higher scores are associated with adverse clinical outcomes is essential to identify scales with the strongest predictive value and refine clinical application; the potential to be effectively integrated into clinical practice and to guide prescribing decisions. For example, Tiisanoja et al. [58] showed scales incorporating multiple biological assays may better reflect peripheral anticholinergic activity [35, 59], with a strong association with hyposalivation [23, 24, 34, 35]. Continued clinical assessment will strengthen clinical confidence, guide safer prescribing, and ultimately improve patient outcomes [15].

The effectiveness of AChB scales in clinical practice also depends on their accessibility and ease of use. Clinical awareness and confidence—most notably within community settings—remain limited [11], highlighting the need for wider integration across healthcare teams. This is particularly important for older adults who are disproportionately affected by inadequate medication reviews and would benefit most from these tools [11].

Despite this, only three scales provide practical guidance alongside AChB scoring [28, 36, 43], and the clinical value of such guidance remains unproven. Emerging evidence suggests simply informing clinicians of a patient's AChB score can prompt meaningful clinical interventions, including deprescribing [60], and may improve patient outcomes [6]. Further research is required to determine the most effective and sustainable approaches, particularly given the heterogeneity among existing scales.

Emerging tools are working to overcome the issues in AChB scale development, including inconsistent drug listings, limited global applicability, and maintaining relevance as new medicines are introduced. For example, Fleetwood et al. [61] developed a novel, web‐based AChB scale that integrates data from multiple previous systems with large pharmacological databases such as Binding DB. This approach enables real‐time updates as new medications become available, improving the scale's relevance and applicability across diverse healthcare settings.

Further research should explore the integration of objective biomarkers, such as blood acetylcholinesterase levels, which show promise for identifying anticholinergic activity [62]. Additionally, access to patient‐specific data, including electronic health records and genetic profiles, could help address inter‐individual variability in drug response, while molecular properties and receptor binding affinities can inform factors such as ACH potency and BBB permeability, supporting a more clinically accurate measure of AChB [62, 63].

This systematic review used a comprehensive literature search with predefined inclusion and exclusion criteria, identifying scales not incorporated into previous reviews [30, 31]. A systematic approach was used to assess scale quality, methodological development, and scoring heterogeneity. Limitations include the equal weighting of all scales in the quality assessment, despite differences in development rigor and validation. The citation analysis of clinical outcome studies was not exhaustive; however, the studies identified were considered sufficient to provide a representative overview of the available evidence. Additional citations were unlikely to contribute substantial new knowledge as no new studies were identified after extensive searching. Moreover, a small number of full texts could not be retrieved despite contacting study authors. The quality assessment tool was developed specifically for AChB scales and has not undergone external validation. Presently, no such tool exists so we developed a bespoke tool based on our extensive experience and expertise in the field. Future reviews could include a formal assessment of inter‐scale disagreement to provide a more comprehensive analysis, and a standardized quality assessment tool for AChB scales could be validated to strengthen future scale evaluation.

## 6 Conclusion

This systematic review identified 21 AChB scales, each showing substantial variation in medication listings, scoring, and development methods. Factors underlying scale heterogeneity include the geographical location and time of scale development, the criteria used for medication inclusion, the consideration of clinical and pharmacological properties, and the approaches used to assign AChB rankings.

This review offers clinicians and researchers a comprehensive overview of current AChB scales and the methodological factors driving their inconsistency. Greater understanding of these elements can guide the development of future tools, with greater accessibility, clinical relevance, and global applicability.

No gold‐standard scale currently exists. However, robust methodological practices—such as mixed method approaches, incorporating dosage, BBB permeability, expert input, and comprehensive drug coverage—as seen in the more novel scale development [61], offer useful direction for future scale refinement. A universally applicable, regularly updated and clinically accessible AChB scale remains needed. Ongoing evaluation of how these scales can predict clinical outcomes will be crucial to optimize their clinical relevance, support safer prescribing, and improve patient care.

## Author Contributions

Phyo K. Myint conceptualized and initiated this review. Orla Vennard and Mansi Tola conducted the literature review and the initial search strategy. All authors contributed to the screening and selection of studies, as well as data extraction. All authors were involved in drafting of the manuscript and the editing process. All authors approved the final version. The authors would like to thank Samual Neil for his contribution to the initiation of the systematic review.

## Funding

The authors have nothing to report.

## Disclosure

A version of the manuscript was previously submitted to *Age and Ageing* and was not accepted for publication. This manuscript is currently not under consideration elsewhere and has not been published previously.

## Ethics Statement

The authors have nothing to report.

## Conflicts of Interest

The authors declare no conflicts of interest.

## Supporting information


**Figure S1:** Peripheral effects of ACH medication.
**Figure S2:** The methodological development of AChB scales.
**Table S1:** Preferred reporting items for systematic reviews and meta‐analyses (PRISMA) checklist.
**Table S2:** Full ovid MEDLINE search strategy.
**Table S3:** Full ovid EMBASE search strategy.
**Table S4:** Full ovid PsycINFO search strategy.
**Table S5:** Quality assessment questions.
**Table S6:** Methodological development of AChB scales.
**Table S7:** AChB scales and their clinical outcome papers.

## References

[1] J. Mur , S. R. Cox , R. E. Marioni , G. Muniz‐terrera , and T. C. Russ , “Increase in Anticholinergic Burden From 1990 to 2015: Age‐Period‐Cohort Analysis in UK Biobank,” British Journal of Clinical Pharmacology 88, no. 3 (2021): 983–993, 10.1111/bcp.15045. 34409635

[2] E. Braithwaite , O. M. Todd , A. Atkin , et al., “Interventions for Reducing Anticholinergic Medication Burden in Older Adults—A Systematic Review and Meta‐Analysis,” Age and Ageing 52, no. 9 (2025): afad176, 10.1093/ageing/afad176. PMC10517713 37740900

[3] J. A. Lieberman , “Managing Anticholinergic Side Effects,” Primary Care Companion to the Journal of Clinical Psychiatry 6, no. Suppl 2 (2004): 20–23. PMC487008 16001097

[4] P. S. Nishtala , S. W. Narayan , T. Wang , and S. N. Hilmer , “Associations of Drug Burden Index With Falls, General Practitioner Visits, and Mortality in Older People,” Pharmacoepidemiology and Drug 23, no. 7 (2014): 753–758, 10.1002/pds.3624. 24723335

[5] S. L. Gray , M. L. Anderson , S. Dublin , et al., “Cumulative Use of Strong Anticholinergics and Incident Dementia,” JAMA Internal Medicine 175, no. 3 (2015): 401–407, 10.1001/jamainternmed.2014.7663. 25621434 PMC4358759

[6] S. N. Hilmer and D. Gnjidic , “The Anticholinergic Burden: From Research to Practice,” Australian Prescriber 45, no. 4 (2022): 118–120, 10.18773/austprescr.2022.031. 36110165 PMC9427617

[7] Prescquipp Community Interest Company , “b140‐Anticholinergics‐Drugs‐21,” 2016.

[8] C. Fox , K. Richardson , I. D. Maidment , et al., “Anticholinergic Medication Use and Cognitive Impairment in the Older Population: The Medical Research Council Cognitive Function and Ageing Study,” Journal of the American Geriatrics Society 59, no. 8 (2011): 1477–1483, 10.1111/j.1532-5415.2011.03491.x. 21707557

[9] N. Cebron Lipovec , J. Jazbar , and M. Kos , “Anticholinergic Burden in Children, Adults and Older Adults in Slovenia: A Nationwide Database Study,” Scientific Reports 10, no. 1 (2020): 9337, 10.1038/s41598-020-65989-9. 32518392 PMC7283335

[10] Á. Tristancho‐Pérez , Á. Villalba‐Moreno , M. D. Santos‐Rubio , et al., “Concordance Among 10 Different Anticholinergic Burden Scales in At‐Risk Older Populations,” Journal of Patient Safety 18 (2012): e816–e821. 10.1097/PTS.0000000000000929 PMC9162063 34693926

[11] G. Araklitis , G. Thiagamoorthy , J. Hunter , A. Rantell , D. Robinson , and L. Cardozo , “Anticholinergic Prescription: Are Healthcare Professionals the Real Burden?,” International Urogynecology Journal 28, no. 8 (2017): 1249–1256, 10.1007/s00192-016-3258-3. 28091711

[12] M. S. Salahudeen , S. N. Hilmer , and P. S. Nishtala , “Comparison of Anticholinergic Risk Scales and Associations With Adverse Health Outcomes in Older People,” Journal of the American Geriatrics Society 63, no. 1 (2015): 85–90, 10.1111/jgs.13206. 25597560

[13] G. Lozano‐Ortega , K. M. Johnston , A. Cheung , et al., “A Review of Published Anticholinergic Scales and Measures and Their Applicability in Database Analyses,” Archives of Gerontology and Geriatrics 87 (2019): 103885, 10.1016/j.archger.2019.05.010. 31155228

[14] A. Lisibach , V. Benelli , M. G. Ceppi , K. Waldner‐Knogler , C. Csajka , and M. Lutters , “Quality of Anticholinergic Burden Scales and Their Impact on Clinical Outcomes: A Systematic Review,” European Journal of Clinical Pharmacology 77 (2020): 147–162, 10.1007/s00228-020-02994. 33011824 PMC7803697

[15] V. M. Srikartika , N. Ha , D. Youens , and R. Moorin , “Assessing the Feasibility of Anticholinergic Burden Scales and Measures in Administrative Data: A Systematic Review,” Archives of Gerontology and Geriatrics 129 (2025): 105646. 39388728 10.1016/j.archger.2024.105646

[16] R. DiazAcedo , A. M. VillalbaMoreno , B. SantosRamos , and S. SanchezFidalgo , “Systematic Review on the Use of Anticholinergic Scales in Elderly Chronic Patients,” Research in Social & Administrative Pharmacy: RSAP 21, no. 3 (2024): 117–133. 39710558 10.1016/j.sapharm.2024.12.004

[17] B. H. Khalil , B. N. Keshk , B. A. Khan , S. Gao , S. H. Khan , and N. C. Pharmd , “Community‐Based Anticholinergic Exposure and Delirium: A Systematic Review,” Delirium (2025), 10.56392/001c.141069.

[18] E. A. Gebreyohannes , B. S. Shibe , W. A. Taye , et al., “Anticholinergic Burden and Health‐Related Quality of Life Among Adult Patients in a Resource‐Limited Setting: A Cross‐Sectional Study,” International Journal of Clinical Pharmacy 46, no. 6 (2024): 1352–1361, 10.1007/s11096-024-01769-z. 39007992 PMC11576878

[19] N. Vidal , E. BrunetGouet , S. Frileux , et al., “Comparative Analysis of Anticholinergic Burden Scales to Explain Iatrogenic Cognitive Impairment and Self‐Reported Side Effects in the Euthymic Phase of Bipolar Disorders: Results From the FACE‐BD Cohort,” European Neuropsychopharmacology 77 (2023): 67–79. 37741163 10.1016/j.euroneuro.2023.08.502

[20] C. Stewart , M. Taylor‐Rowan , R. L. Soiza , T. J. Quinn , Y. K. Loke , and P. K. Myint , “Anticholinergic Burden Measures and Older People's Falls Risk: A Systematic Prognostic Review,” Therapeutic Advances in Drug Safety 12 (2021): 20420986211016645, 10.1177/20420986211016645. 34104401 PMC8170331

[21] G. O. Phutietsile , N. Fotaki , H. A. Jamieson , and P. S. Nishtala , “The Association Between Anticholinergic Burden and Mobility: A Systematic Review and Meta‐Analyses,” BMC Geriatrics 23, no. 1 (2023): 161. 36949391 10.1186/s12877-023-03820-6 PMC10035151

[22] B. Bell and A. Avery , “Identifying Anticholinergic Burden in Clinical Practice,” Prescriber 32, no. 3 (2021): 20, 10.1002/psb.1901.

[23] U. Ehrt , K. Broich , J. P. Larsen , C. Ballard , and D. Aarsland , “Use of Drugs With Anticholinergic Effect and Impact on Cognition in Parkinson's Disease: A Cohort Study,” Journal of Neurology, Neurosurgery & Psychiatry 81, no. 2 (2009): 160, 10.1136/jnnp.2009.186239. 19770163

[24] M. L. Chew , B. H. Mulsant , B. G. Pollock , et al., “Anticholinergic Activity of 107 Medications Commonly Used by Older Adults,” Journal of the American Geriatrics Society 56, no. 7 (2008): 1333–1341, 10.1111/j.1532-5415.2008.01737.x. 18510583

[25] L. M. Kalisch Ellett , N. L. Pratt , E. N. Ramsay , J. D. Barratt , and E. E. Roughead , “Multiple Anticholinergic Medication Use and Risk of Hospital Admission for Confusion or Dementia,” Journal of the American Geriatrics Society 62, no. 10 (2014): 1916–1922, 10.1111/jgs.13054. 25284144

[26] T. T. Klamer , M. Wauters , M. Azermai , et al., “A Novel Scale Linking Potency and Dosage to Estimate Anticholinergic Exposure in Older Adults: The Muscarinic Acetylcholinergic Receptor ANTagonist Exposure Scale,” Basic & Clinical Pharmacology & Toxicology 120, no. 6 (2017): 582, 10.1111/bcpt.12699. 28090742

[27] W. K. Summers , “A Clinical Method of Estimating Risk of Drug Induced Delirium,” Life Sciences 22 (1978): 1511–1516. 672410 10.1016/0024-3205(78)90006-1

[28] D. Sumukadas , M. E. T. Mcmurdo , A. A. Mangoni , and B. Guthrie , “Temporal Trends in Anticholinergic Medication Prescription in Older People: Repeated Cross‐Sectional Analysis of Population Prescribing Data,” Age and Ageing 43, no. 4 (2013): 515, 10.1093/ageing/aft199. 24334709

[29] J. Briet , H. Javelot , E. Heitzmann , et al., “The Anticholinergic Impregnation Scale: Towards the Elaboration of a Scale Adapted to Prescriptions in French Psychiatric Settings,” Thérapie 72, no. 4 (2017): 427, 10.1016/j.therap.2016.12.010. 28336159

[30] C. M. B. Kehman , M. Schlunsen , and L. J. Kjeldsen , “Categorisation of Patients' Anticholinergic Burden at Admission and Discharge From the Geriatric Ward of Sonderjylland Hospital,” Pharmacy 12, no. 6 (2024): 160. 39585086 10.3390/pharmacy12060160 PMC11587422

[31] F. Mizokami , T. Mizuno , R. Taguchi , et al., “Development of the Japanese Anticholinergic Risk Scale: English Translation of the Japanese Article,” Geriatrics & Gerontology International 25 (2024): 5–13. 39635941 10.1111/ggi.15001 PMC11711069

[32] H. Ramos , L. Moreno , J. Pérez‐Tur , C. Cháfer‐Pericás , G. García‐Lluch , and J. Pardo , “CRIDECO Anticholinergic Load Scale: An Updated Anticholinergic Burden Scale. Comparison With the ACB Scale in Spanish Individuals With Subjective Memory Complaints,” JPM 12, no. 2 (2022): 207, 10.3390/jpm12020207. 35207695 PMC8876932

[33] J. L. Rudolph , M. J. Salow , M. C. Angelini , and R. E. McGlinchey , “The Anticholinergic Risk Scale and Anticholinergic Adverse Effects in Older Persons,” Archives of Internal Medicine 168, no. 5 (2008): 508–513, 10.1001/archinternmed.2007.106. 18332297

[34] R. M. Carnahan , B. C. Lund , P. J. Perry , B. G. Pollock , and K. R. Culp , “The Anticholinergic Drug Scale as a Measure of Drug‐Related Anticholinergic Burden: Associations With Serum Anticholinergic Activity,” Journal of Clinical Pharma 46, no. 12 (2006): 1481, 10.1177/0091270006292126. 17101747

[35] M. L. Ancelin , S. Artero , F. Portet , A. Dupuy , J. Touchon , and K. Ritchie , “Non‐Degenerative Mild Cognitive Impairment in Elderly People and Use of Anticholinergic Drugs: Longitudinal Cohort Study,” BMJ 332, no. 7539 (2006): 455, 10.1136/bmj.38740.439664.de. 16452102 PMC1382539

[36] M. Boustani , N. Campbell , S. Munger , I. Maidment , and C. Fox , “Impact of Anticholinergics on the Aging Brain: A Review and Practical Application,” Aging Health 4, no. 3 (2008): 311, 10.2217/1745509x.4.3.311.

[37] R. T. Nery and A. M. M. Reis , “Development of a Brazilian Anticholinergic Activity Drug Scale,” Einstein (Sao Paulo) 17, no. 2 (2019): eAO4435. 30942279 10.31744/einstein_journal/2019AO4435 PMC6443211

[38] S. Yamada , M. Mochizuki , J. Chimoto , R. Futokoro , S. Kagota , and K. Shinozuka , “Development of a Pharmacological Evidence‐Based Anticholinergic Burden Scale for Medications Commonly Used in Older Adults,” Geriatrics & Gerontology International 23, no. 7 (2023): 558, 10.1111/ggi.14619. 37313633 PMC11503540

[39] T. Rube , A. Ecorcheville , E. Londos , S. Modig , and P. Johansson , “Development of the Swedish Anticholinergic Burden Scale (Swe‐ABS),” BMC Geriatrics 23, no. 1 (2023): 518, 10.1186/s12877-023-04225-1. 37626293 PMC10464171

[40] G. Sittironnarit , D. Ames , A. I. Bush , et al., “Effects of Anticholinergic Drugs on Cognitive Function in Older Australians: Results From the AIBL Study,” Dementia and Geriatric Cognitive Disorders 31, no. 3 (2025): 173, 10.1159/000325171. 21389718

[41] E. K. Kiesel , Y. M. Hopf , and M. Drey , “An Anticholinergic Burden Score for German Prescribers: Score Development,” BMC Geriatrics 18, no. 1 (2018): 239, 10.1186/s12877-018-0929-6. 30305048 PMC6180424

[42] K. Jun , S. Hwang , Y. Ah , Y. Suh , and J. Lee , “Development of an Anticholinergic Burden Scale Specific for Korean Older Adults,” Geriatrics & Gerontology International 19, no. 7 (2019): 628, 10.1111/ggi.13680. 31033150

[43] D. Bishara , D. Harwood , J. Sauer , and D. M. Taylor , “Anticholinergic Effect on Cognition (AEC) of Drugs Commonly Used in Older People,” International Journal of Geriatric Psychiatry 32, no. 6 (2016): 650, 10.1002/gps.4507. 27280553

[44] L. Andre , A. Gallini , F. Montastruc , et al., “Anticholinergic Exposure and Cognitive Decline in Older Adults: Effect of Anticholinergic Exposure Definitions in a 3‐Year Analysis of the Multidomain Alzheimer Preventive Trial (MAPT) Study,” British Journal of Clinical Pharmacology 85, no. 1 (2018): 71, 10.1111/bcp.13734. 30098049 PMC6303211

[45] D. Bishara , G. Perera , D. Harwood , et al., “The Anticholinergic Effect on Cognition (AEC) Scale—Associations With Mortality, Hospitalisation and Cognitive Decline Following Dementia Diagnosis,” International Journal of Geriatric Psychiatry 35, no. 9 (2020): 1069, 10.1002/gps.5330. 32394521

[46] T. S. Dinh , A. D. Meid , H. Rudolf , et al., “Anticholinergic Burden Measures, Symptoms, and Fall‐Associated Risk in Older Adults With Polypharmacy: Development and Validation of a Prognostic Model,” PLoS One 18, no. 1 (2023): e0280907, 10.1371/journal.pone.0280907. 36689445 PMC9870119

[47] A. Ziad , R. Olekhnovitch , F. Ruiz , et al., “Anticholinergic Drug Use and Cognitive Performances in Middle Age: Findings From the CONSTANCES Cohort,” Journal of Neurology, Neurosurgery, and Psychiatry 89, no. 10 (2018): 1107, 10.1136/jnnp-2018-318190. 30196250 PMC6166611

[48] M. Richards , “Extent and Causes of International Variations in Drug Usage,” 2010 accessed March 30, 2025, https://assets.publishing.service.gov.uk/government/uploads/system/uploads/attachment_data/file/216249/dh_117977.pdf.

[49] O. Holdgate , “Comparing New Medicine Availability Across Europe,” The Association of the British Pharmaceutical Industry, accessed March 30, 2025, https://www.abpi.org.uk/media/blogs/2024/june/comparing‐new‐medicine‐availability‐across‐europe/.

[50] World Health Organisation , Medication Safety in Polypharmacy (World Health Organisation, 2019), 11–15, https://www.who.int/docs/default‐source/patient‐safety/who‐uhc‐sds‐2019‐11‐eng.pdf.

[51] A. R. Green , L. M. Reifler , E. A. Bayliss , L. A. Weffald , and C. M. Boyd , “Drugs Contributing to Anticholinergic Burden and Risk of Fall or Fall‐Related Injury Among Older Adults With Mild Cognitive Impairment, Dementia and Multiple Chronic Conditions: A Retrospective Cohort Study,” Drugs & Aging 36, no. 3 (2020): 289, 10.1007/s40266-018-00630-z. PMC6386184 30652263

[52] K. Gupta , K. Kaur , B. S. Aulakh , and S. Kaushal , “Fesoterodine for Overactive Bladder: A Review of the Literature,” 71, no. 5 (2010): 273–288. 10.1016/j.curtheres.2010.10.003 PMC3969610 24688149

[53] A. J. E. G. Berger , E. M. Dukes , J. Edelsberg , B. R. Stacey , and G. Oster , “Use of Tricyclic Antidepressants in Older Patients With Painful Neuropathies,” European Journal of Clinical Pharmacology 62, no. 9 (2006): 757–764, 10.1007/s00228-006-0161-8. 16802165

[54] K. M. Rudd , C. L. Raehl , C. A. Bond , T. J. Abbruscato , and A. C. Stenhouse , “Methods for Assessing Drug‐Related Anticholinergic Activity,” Pharmacotherapy 25, no. 11 (2005): 1592, 10.1592/phco.2005.25.11.1592. 16232021

[55] S. B. Al Rihani , M. Deodhar , L. I. Darakjian , et al., “Quantifying Anticholinergic Burden and Sedative Load in Older Adults With Polypharmacy: A Systematic Review of Risk Scales and Models,” Drugs & Aging 38, no. 11 (2021): 977–994, 10.1007/s40266-021-00895-x. 34751922 PMC8592980

[56] A. Lisibach , G. Gallucci , P. E. Beeler , C. Csajka , and M. Lutters , “High Anticholinergic Burden at Admission Associated With In‐Hospital Mortality in Older Patients: A Comparison of 19 Different Anticholinergic Burden Scales,” Basic & Clinical Pharmacology & Toxicology 130, no. 2 (2021): 288, 10.1111/bcpt.13692. 34837340 PMC9299782

[57] H. Kersten , E. Molden , I. K. Tolo , E. Skovlund , K. Engedal , and T. B. Wyller , “Cognitive Effects of Reducing Anticholinergic Drug Burden in a Frail Elderly Population: A Randomized Controlled Trial,” Journals of Gerontology. Series A, Biological Sciences and Medical Sciences 68, no. 3 (2013): 271–278, 10.1093/gerona/gls176. 22982689

[58] A. Tiisanoja , A.‐H. Syrjälä , A. Kullaa , and P. Ylöstalo , “Anticholinergic Burden and Dry Mouth in Middle‐Aged People,” JDR Clinical & Translational Research 5, no. 1 (2019): 62, 10.1177/2380084419844511. 31013461

[59] H. Kersten , E. Molden , T. Willumsen , K. Engedal , and T. B. Wyller , “Higher Anticholinergic Drug Scale (ADS) Scores Are Associated With Peripheral but Not Cognitive Markers of Cholinergic Blockade. Cross Sectional Data From 21 Norwegian Nursing Homes,” British Journal of Clinical Pharmacology 75, no. 3 (2012): 842, 10.1111/j.1365-2125.2012.04411. PMC3575951 22924454

[60] H. S. Tay , R. L. Sozia , and A. A. Mangoni , “Minimizing Anticholinergic Drug Prescribing in Older Hospitalized Patients: A Full Audit Cycle,” Therapeutic Advances in Drug Safety 5, no. 3 (2024): 121–128, 10.1177/2042098614523638. PMC4110854 25083267

[61] C. Fleetwood , M. Salehi , R. Ward , et al., A Novel Machine Learning Approach to Anticholinergic Burden Quantification (University of Kent, Academic Repository, 2021).

[62] J. López‐Álvarez , J. Sevilla‐Llewellyn‐Jones , and L. Agüera‐Ortiz , “Anticholinergic Drugs in Geriatric Psychopharmacology,” Frontiers in Neuroscience 13 (2019): 1309, 10.3389/fnins.2019.01309. 31866817 PMC6908498

[63] M. V. Relling and W. E. Evans , “Pharmacogenomics in the Clinic,” Nature 526, no. 7573 (2015): 343–350, 10.1038/nature15817. 26469045 PMC4711261

