# Supplementary material for: Anticholinergic Medication Burden Scales: A Systematic Review
Source: J Am Geriatr Soc. 2026 Feb 23;74(6):1771–84. doi: 10.1111/jgs.70352 (PMC13266442; doi:10.1111/jgs.70352)
Supplement: Supplementary file 1 — Figure S1: Peripheral effects of ACH medication. Figure S2: The methodological development of AChB scales. Table S1: Preferred reporting items for systematic reviews and meta‐analyses (PRISMA) checklist. Table S2: Full ovid MEDLINE search strategy. Table S3: Full ovid EMBASE search strategy. Table S4: Full ovid PsycINFO search strategy. Table S5: Quality assessment questions. Table S6: Methodological development of AChB scales. Table S7: AChB scales and their clinical outcome papers. [file JGS-74-1771-s001.pdf]

Anticholinergic Medication Burden Scales:

A Systematic Review

Supplementary Data

Table of Contents

Supplementary Figure 1. 2

Supplementary Figure 2. 3

Supplementary Table1. Preferred Reporting Items for Systematic Reviews and Meta-Analyses (PRISMA) Checklist..4

Supplementary Table 2. Full Ovid MEDLINE Search Strategy..6

Supplementary Table 3. Full Ovid EMBASE Search Strategy 6

Supplementary Table 4. Full Ovid PsycINFO Search Strategy 7

Supplementary Table 5. Quality Assessment Questions. 8

Supplementary Table 6. Methodological Development of AChB Scales...........................9

Supplementary Table 7. AChB Scales and their Clinical Outcome Papers 11

Supplementary Figure 1.

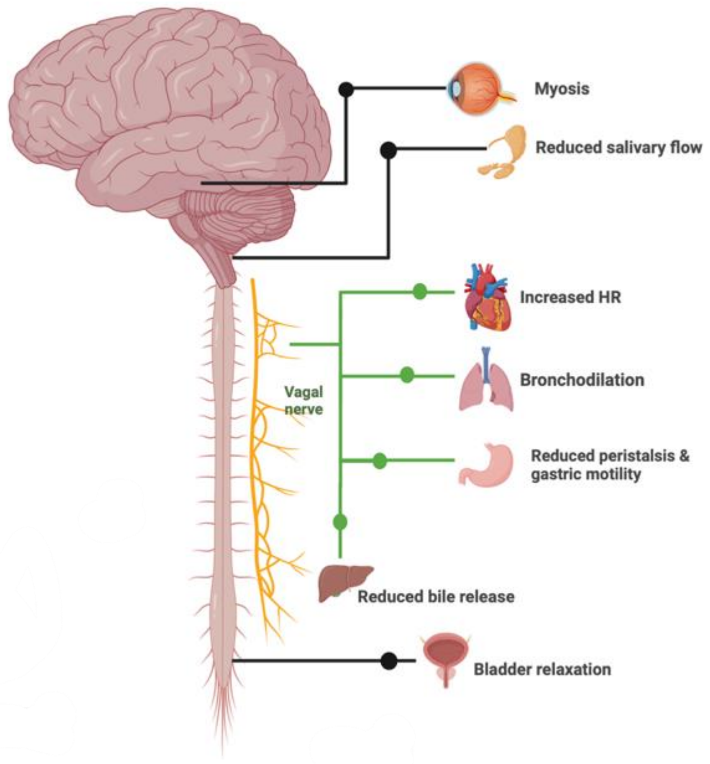

Supplementary figure 1. Peripheral Effects of ACH Medication

Anticholinergic medication inhibits the neurotransmitter acetylcholine from binding to its target receptors. This results in numerous central and peripheral effects.. The peripheral effects of ACH medication are illustrated above. ACH = anticholinergic. Created in https://BioRender.com.

Supplementary Figure 2.

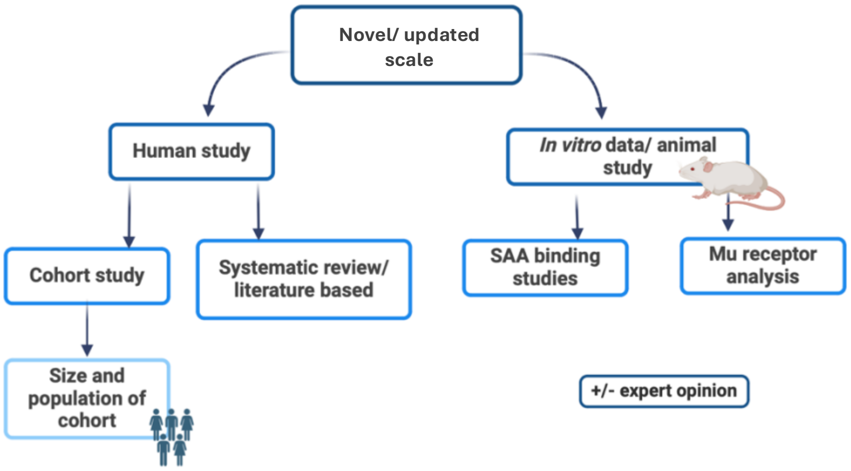

Supplementary Figure 2. The Methodological Development of AChB Scales.

Flow diagram highlighting the common methodological steps taken during the development of an AChB scale. This outlines the methods used to select included medication and determine their AChB rankings. The main types of methodologies include human studies or those based on in vitro / animal data. Scales can be developed from cohort studies (with the population studied influencing the score outcome) previous literature, SAA studies, muscarinic receptor analysis, alongside a combination of techniques. Many scales use expert opinion to provide the final ranking of the medication. SAA= Serum Anticholinergic Activity, Mu= Muscarinic, AChB= Anticholinergic burden.
Created in. https://BioRender.com.

Supplementary Table1. Preferred Reporting Items for Systematic Reviews and Meta-Analyses (PRISMA) Checklist

Section and Topic	Item #	Checklist item	Location where item is reported
**TITLE**			
Title	1	Identify the report as a systematic review.	Page 1
**ABSTRACT**			
Abstract	2	See the PRISMA 2020 for Abstracts checklist.	Page 3
**INTRODUCTION**			
Rationale	3	Describe the rationale for the review in the context of existing knowledge.	Page 7-19
Objectives	4	Provide an explicit statement of the objective(s) or question(s) the review addresses.	Page 20
**METHODS**			
Eligibility criteria	5	Specify the inclusion and exclusion criteria for the review and how studies were grouped for the syntheses.	Page 22
Information sources	6	Specify all databases, registers, websites, organisations, reference lists and other sources searched or consulted to identify studies. Specify the date when each source was last searched or consulted.	Page 21
Search strategy	7	Present the full search strategies for all databases, registers and websites, including any filters and limits used.	Page 21
Selection process	8	Specify the methods used to decide whether a study met the inclusion criteria of the review, including how many reviewers screened each record and each report retrieved, whether they worked independently, and if applicable, details of automation tools used in the process.	Page 23
Data collection process	9	Specify the methods used to collect data from reports, including how many reviewers collected data from each report, whether they worked independently, any processes for obtaining or confirming data from study investigators, and if applicable, details of automation tools used in the process.	Page 23
Data items	10a	List and define all outcomes for which data were sought. Specify whether all results that were compatible with each outcome domain in each study were sought (e.g. for all measures, time points, analyses), and if not, the methods used to decide which results to collect.	Page 23
	10b	List and define all other variables for which data were sought (e.g. participant and intervention characteristics, funding sources). Describe any assumptions made about any missing or unclear information.	N/A
Study risk of bias assessment	11	Specify the methods used to assess risk of bias in the included studies, including details of the tool(s) used, how many reviewers assessed each study and whether they worked independently, and if applicable, details of automation tools used in the process.	Page 23-24
Effect measures	12	Specify for each outcome the effect measure(s) (e.g. risk ratio, mean difference) used in the synthesis or presentation of results.	N/A
Synthesis methods	13a	Describe the processes used to decide which studies were eligible for each synthesis (e.g. tabulating the study intervention characteristics and comparing against the planned groups for each synthesis (item #5)).	Page 23-24
	13b	Describe any methods required to prepare the data for presentation or synthesis, such as handling of missing summary statistics, or data conversions.	Page 23-24
	13c	Describe any methods used to tabulate or visually display results of individual studies and syntheses.	Page 23-24
	13d	Describe any methods used to synthesize results and provide a rationale for the choice(s). If meta-analysis was performed, describe the model(s), method(s) to identify the presence and extent of	Page 23-24

Section and Topic	Item #	Checklist item	Location where item is reported
		statistical heterogeneity, and software package(s) used.	
	13e	Describe any methods used to explore possible causes of heterogeneity among study results (e.g. subgroup analysis, meta-regression).	Page 23-24
	13f	Describe any sensitivity analyses conducted to assess robustness of the synthesized results.	N/A
Reporting bias assessment	14	Describe any methods used to assess risk of bias due to missing results in a synthesis (arising from reporting biases).	N/A
Certainty assessment	15	Describe any methods used to assess certainty (or confidence) in the body of evidence for an outcome.	N/A
**RESULTS**			
Study selection	16a	Describe the results of the search and selection process, from the number of records identified in the search to the number of studies included in the review, ideally using a flow diagram.	Page 26
	16b	Cite studies that might appear to meet the inclusion criteria, but which were excluded, and explain why they were excluded.	Page 27
Study characteristics	17	Cite each included study and present its characteristics.	Page 28
Risk of bias in studies	18	Present assessments of risk of bias for each included study.	Page 32
Results of individual studies	19	For all outcomes, present, for each study: (a) summary statistics for each group (where appropriate) and (b) an effect estimate and its precision (e.g. confidence/credible interval), ideally using structured tables or plots.	N/A
Results of syntheses	20a	For each synthesis, briefly summarise the characteristics and risk of bias among contributing studies.	N/A
	20b	Present results of all statistical syntheses conducted. If meta-analysis was done, present for each the summary estimate and its precision (e.g. confidence/credible interval) and measures of statistical heterogeneity. If comparing groups, describe the direction of the effect.	N/A
	20c	Present results of all investigations of possible causes of heterogeneity among study results.	N/A
	20d	Present results of all sensitivity analyses conducted to assess the robustness of the synthesized results.	N/A
Reporting biases	21	Present assessments of risk of bias due to missing results (arising from reporting biases) for each synthesis assessed.	N/A
Certainty of evidence	22	Present assessments of certainty (or confidence) in the body of evidence for each outcome assessed.	N/A
**DISCUSSION**			
Discussion	23a	Provide a general interpretation of the results in the context of other evidence.	Page 37-46
	23b	Discuss any limitations of the evidence included in the review.	Page 46
	23c	Discuss any limitations of the review processes used.	Page 46
	23d	Discuss implications of the results for practice, policy, and future research.	Page 47
**OTHER INFORMATION**			
Registration and protocol	24a	Provide registration information for the review, including register name and registration number, or state that the review was not registered.	Page 21
	24b	Indicate where the review protocol can be accessed, or state that a protocol was not prepared.	Page 21
	24c	Describe and explain any amendments to information provided at registration or in the protocol.	N/A
Support	25	Describe sources of financial or non-financial support for the review, and the role of the funders or sponsors in the review.	N/A
Competing interests	26	Declare any competing interests of review authors.	N/A
Availability of data, code and other materials	27	Report which of the following are publicly available and where they can be found: template data collection forms; data extracted from included studies; data used for all analyses; analytic code; any other materials used in the review.	N/A

Supplementary Table 2. Full Ovid MEDLINE Search Strategy

Line	Search term
1.	anticholinergic*.mp. OR anti-cholinergic*.mp. OR cholinergic antagonist*.mp. OR antimuscarinic*.mp. OR anti-muscarinic*.mp. OR muscarinic antagonist*.mp.
2.	scale*.mp. OR score*.mp. OR rank*.mp. OR rating*.mp. OR grading*.mp. OR index*.mp. OR classification*.mp.
3.	1 AND 2
4.	Limit 3 to humans

Note: .mp. = multi-purpose

Supplementary Table 3. Full Ovid EMBASE Search Strategy

Line	Search term
1.	anticholinergic*.tw. OR anti-cholinergic*.tw. OR cholinergic antagonist*.tw. OR antimuscarinic*.tw. OR anti-muscarinic*.tw. OR muscarinic antagonist*.tw.
2.	scale*.tw. OR score*.tw. OR rank*.tw. OR rating*.tw. OR grading*.tw. OR index*.tw. OR classification*.tw.
3.	1 AND 2
4.	Limit 3 to humans

Note: .tw. = text word

Supplementary Table 4. Full Ovid PsycINFO Search Strategy

Line	Search term
1.	anticholinergic*.mp. OR anti-cholinergic*.mp. OR cholinergic antagonist*.mp. OR antimuscarinic*.mp. OR anti-muscarinic*.mp. OR muscarinic antagonist*.mp.
2.	scale*.mp. OR score*.mp. OR rank*.mp. OR rating*.mp. OR grading*.mp. OR index*.mp. OR classification*.mp.
3.	1 AND 2
4.	Limit 3 to humans

Note: .tw. = text word

Supplementary Table 5. Quality Assessment Questions.

1. Is the methodology clearly described?

2. Is the scale based on composite methodology? (e.g. combination of expert opinion, existing literature, *in vitro* data etc.)

   a. If literature-based, was an appropriate systematic literature search described?

3. Does the scale include expert opinion?

   a. How many experts? (≥3 = good, 2 = fair, 1 = poor)

   b. Were the experts multidisciplinary?

   c. What method was used for experts to reach a consensus (e.g. Delphi method etc.)

   d. Is the method for resolving disagreements between experts reported and appropriate?

4. Does the scale take into account the medication dose?

5. Does the scale take into account the duration of medication use?

6. Does the scale take into account all routes of medication administration?

7. Was the scale designed with specific outcome(s) in mind?

8. Does the scale subsequently show positive association with the intended specific outcome(s)?

Supplementary Table 6. Methodological Development of AChB Scales.

Anticholinergic medication scale	Methodology Step 1	Step 2	Step 3	Step 4
**Summers list** [1]	Cohort drug list (84 individuals)	Interview of participants	Calculation of drug exposure, considering daily effective dose and drug class	
**ABC** [2]	Cohort drug list (372 individuals)	Interview of participants	Literature review of SAA levels	Ranking by expert committee
**ADS** [3]	Cohort drug list (201 individuals)	SAA blood draw	Dosage estimation using maximum recommended daily dose, for dose adjusted-ADS score.	
**ARS** [4]	Cohort drug list (249 individuals)	Expert team review, based on published literature, including receptor binding data.	Ranking of medication by expert committee.	
**ACB** [5]	Systematic review.	Expert team review, based on published literature.	Interdisciplinary team scoring drugs	
**Chews list** [6]	Drug list: Most frequently dispensed medications to residents of long-term care facilities	*In vitro* AA, using competitive radioreceptor binding (rodent model)	C_{max}: dose and AA relationship	
**Ehrt** *et al* [7]	Cohort drug list (235 individuals)	Modulate methods by Chew et al	Ranking of medication by expert committee	
**ALS** [8]	Cohort drug list (1,112 individuals)	SAA scores from previous data	Clinician rated ACH scores	
**Ellett's list** [9]	Cohort drug list (220,000 individuals)	Ranking of medication using ARS and ADS	Review of Australian medicine literature.	
**mARS** [10]	Cohort drug list (141,073 individuals) and BNF medication.	Ranking of medication using ARS	Available literature and expert knowledge to score updated Ach drugs	
**AEC** [11]	Review of BNF medication and systematic review	Database search and literature review	Database searched for drugs binding affinities to Mu receptors and radioreceptor binding measurement of AA.	Ranking by expert committee
**AIS** [12]	Cohort drug list (7278 prescriptions)	*In vitro* data on SAA	Reported previously published AChB scores	Clinicians ranked drugs

Study	Methodology			
	**Step 1**	**Step 2**	**Step 3**	**Step 4**
**MARANTE** [13]	Cohort drug list (1258 individuals)	Dosage concepts determined, using health databases and expert guidance	Ach load estimated through potency and dosage calculation	
**ABS** [14]	Systematic review	Medication mechanism of action and ADRs reviewed by researcher	Cohort study (34 participants)	Production of pocket-sized practical guidelines
**KABS** [15]	Systematic review	Expert scoring of drugs	Calculation of drug exposure, considering daily effective dose and drug class	
**BAAS** [16]	Systematic review	Reclassification of drugs from previous scales to fit 3-point scale considering previous rankings, SAA or reported AEs	Literature review of SAA levels	Ranking by expert committee
**CALS** [17]	Systematic review	Expert committee ranked drugs	Dosage estimation using maximum recommended daily dose, for dose adjusted-ADS score.	
**SweABS** [18]	Systematic review	Expert committee ranking based on previous scores, database reported AEs, SAA and Mu binding affinities		
**Kehman** C.M.B. *et al* [19]	Systematic review	APIs systematically evaluated for AA		
**ABS** [20]	Drug list (260 drugs commonly used in the elderly in Japan)	Mu binding affinity (rats) using radioreceptor assay.	C_{max} of drugs was cited after interview form of each drug provided by pharmacological companies	
**JARS** [21]	Systematic review	Scores from the previous published scales where standardised using an algorithm.	Expert committee scored drugs with a score of 3, considering relevant literature of its AEs and mechanism of action	

Supplementary Table 6. *Methodological development steps of 21 studies included in this review, along with the number of individuals included in each cohort, are provided where applicable. ABC= Anticholinergic Burden Classification, ADS= Anticholinergic Drug Scale, ARS= Anticholinergic Risk Scale, ACB=Anticholinergic Cognitive Burden, ACL= Anticholinergic Load Scale, mARS= Modified Anticholinergic Risk Scale, AEC= Anticholinergic Effect on Cognition, AIS= Anticholinergic Impregnation scale, MARANTE= Muscarinic Acetylcholinergic Receptor Antagonist Exposure Scale, KABS=Korean Anticholinergic Burden Scale, BAAS= Brazilian Anticholinergic Activity Drug Scale, CALS=CRIDECO Anticholinergic Loading Scale, SweABS= Swedish Anticholinergic Burden Scale, JARS= Japanese Anticholinergic Risk Scale, Ka= Dissociation constant, SAA= Serum Anticholinergic Activity, AA= Anticholinergic Activity, Ach= Anticholinergic, BBB= Blood Brain Barrier, BNF= British National Formulary, APIs= Active Pharmaceutical Ingredients, Cmax= Peak serum concentration, ADRs= Adverse Drug Reactions, AEs= Adverse Effect, Mu= Muscarinic*

Supplementary Table 7. AChB Scales and their Clinical Outcome Papers

Scale	Clinical Outcome study	Outcome Evaluated against	Outcome of Evaluation	Type of study
**Summers' list** [20]	Internal study	Drug induced delirium	Positive correlation	Cohort study
	Han *et al* [22]	Delirium in older adults	Positive correlation	Cohort study
**ABC** [2]	Internal study	Cognitive function	Positive correlation	Cohort study
	Lisibach *et al* [23]	In hospital mortality and length of stay	Positive correlation	Cohort study
	Tiisanoja *et al* [24]	Hyposalivation	Positive correlation	Cross sectional cohort study
	Salahudeen *et al* [25]	Hospital admissions, hospitalizations for falls, hospital length of stay (LOS), and more visits to general practitioners (GPs).	Positive correlation	Population based study
	Vidal *et al* [26]	Peripheral ACH SEs in BD	Positive correlation	Cohort study
**ADS** [3]	Conti MSB *et al* [27]	Polypharmacy, cognition and functionality	Positive correlation	Cross sectional study
	Michail *et al* [28]	Hyposalivation	Positive correlation	Cross sectional study
	Aldebert G *et al* [29]	Age related macular degeneration	Positive correlation	Case control study
	Andre *et al.*, 2018 [30]	Cognitive decline in older adults	No association	Cohort study
	Salahudeen *et al* [25]	Hospital admissions, hospitalizations for falls, hospital length of stay (LOS), and more visits to general practitioners (GPs).	Positive correlation	Population based study
	Dinh *et al* [31]	Ability to predict falls	Limited value	RTC
	Hanlon *et al* [32]	Cardiovascular events, mortality, admission due to fall/fracture, or admission with dementia/delirium	Positive correlation	Cohort study
	Lisibach *et al* [23]	In hospital mortality and length of stay	Positive correlation	Cohort study
	Kersten H [33]	Cognitive function in the elderly	No correlation	RTC
**ARS** [4]	Internal study	Adverse effects of ACH medication in older adults	Positive correlation	Cohort study
	Conti MSB [27]	Polypharmacy, cognition and functionality	Positive correlation	Cross sectional study
	Andre *et al* [30]	Cognitive decline in older adults	No association	Cohort study
	Hsu *et al* [34]	Emergency department visits, all-cause hospitalizations, fracture-specific hospitalizations, and incident dementia	Positive correlation	Cohort study
	Crispo *et al* [35]	Fracture and delirium incidence in Parkinson disease patients	Positive correlation	Cohort study
	Hanlon *et al* [32]	Cardiovascular events, mortality, admission due to fall/fracture, or admission with dementia/delirium	Positive correlation	Cohort study
	Vidal *et al* [26]	Peripheral ACH SEs in BD	Positive correlation	Cohort study
	Salahudeen *et al* [25]	Hospital admissions, hospitalizations for falls, hospital length of stay (LOS), and more visits to general practitioners (GPs).	Positive correlation	Population based study
**ACB** [5]	Muglia *et al* [36]	Dysphagia in hospitalised patients	Positive correlation	Cohort study
	Conti MSB *et al* [27]	Polypharmacy, cognition and functionality	Positive correlation	Cross sectional study

Scale	Clinical Outcome study	Outcome Evaluated against	Outcome of Evaluation	Type of study
	Mcquaid *et al* [37]	Self-reported cognitive decline in individuals with autism	Positive correlation	Cross sectional study
	Ablett *et al* [38]	Falls in middle aged women	Positive correlation	Cohort study
	Andre *et al* [30]	Cognitive decline in older adults	No association	Cross sectional study
	Tsai *et al* [39]	Cardiovascular events	Positive correlation	Cohort study
	Lisibach *et al* [23]	In hospital mortality and length of stay	Positive correlation	Cross sectional study
	Hsu *et al* [34]	Emergency department visits, all-cause hospitalizations, fracture-specific hospitalizations, and incident dementia	Positive correlation	Cohort study
	Tiisanoja *et al* [24]	Hyposalivation	Positive correlation	Cohort study
	Stenbäck *et al* [40]	Hyposalivation in the elderly	Positive correlation	Cross sectional cohort study
	Ziad *et al* [41]	Cognitive decline in middle-aged adults	No correlation	Population based study
	Vidal *et al* [26]	Peritheral ACH Ses in BD	Positive correlation	Cross sectional study
	Salahudeen *et al* [25]	Hospital admissions, hospitalizations for falls, hospital length of stay (LOS), and more visits to general practitioners (GPs).	Positive correlation	Cohort study
	Hanlon *et al* [32]	Cardiovascular events, mortality, admission due to fall/fracture, or admission with dementia/delirium	Positive correlation	Population based study
	Huang *et al* [42]	Acute cardiovascular events	Positive correlation	Cohort study
**Chew's list** [6]				Case control study
	Lisibach *et al* [23]	In hospital mortality and length of stay	Positive correlation	
	Tiisanoja *et al* [43]	Hyposalvation	Positive correlation	Cohort study
	Salahudeen *et al* [25]	Hospital admissions, hospitalizations for falls, hospital length of stay (LOS), and more visits to general practitioners (GPs).	Positive correlation	Cross sectional cohort study
	Stenbäck *et al* [40]	Hyposalvation in the elderly	Positive correlation	Population based study
	Vidal *et al* [26]	Peripheral ACH SEs in BD	Positive correlation	Population based study
**Ehrt** *et al* [7]				Cohort study
	Internal study	Cognitive decline	Positive correlation	
	Tiisanoja [24]	Hyposalvation	Positive correlation	Cohort study (PD patients)
	Salahudeen *et al* [25]	Hospital admissions, hospitalizations for falls, hospital length of stay (LOS), and more visits to general practitioners (GPs).	Positive correlation	Cross sectional cohort study
	Stenbäck *et al* [40]	Hyposalvation in the elderly	Positive correlation	Population based study
	Hanlon *et al* [32]	Cardiovascular events, mortality, admission due to fall/fracture, or admission with dementia/delirium	Positive correlation	Population based study
**ACL** [8]				
	Internal study	Cognitive decline	Positive correlation	Cohort study
	Lisibach *et al* [23]	In hospital mortality and length of stay	Positive correlation	Cohort study
	Stenbäck *et al* [40]	Hyposalvation in the elderly	Positive correlation	Cohort study
	Salahudeen *et al* [25]	Hospital admissions, hospitalizations for falls, hospital length of stay (LOS), and more visits to general practitioners (GPs).	Positive correlation	Population based study
	Hanlon *et al* [32]	Cardiovascular events, mortality, admission due to fall/fracture, or admission with dementia/delirium	Positive correlation	Population based study

Scale	Clinical Outcome study	Outcome Evaluated against	Outcome of Evaluation	Type of study
**Ellett's list** [9]	Internal study	Risk of hospitalization for confusion or dementia	Positive correlation	Cohort study
**mARS** [10]	Hauff *et al* [44]	Fracture risk in older adults	Positive correlation	Cohort study
	Hanlon *et al* [32]	Cardiovascular events, mortality, admission due to fall/fracture, or admission with dementia/delirium	Positive correlation	Cohort study
	Clarke *et al* [45]	Physical activity in older adults	Positive correlation	Cohort study
	Vidal *et al* [26]	Peripheral ACH SEs in BD	Positive correlation	Cohort study
**AEC** [30]	Vidal, Nathan *et al* [46]	Cognitive impairment in Schizophrenia	Positive correlation	Cross sectional study
	Hanlon *et al* [32]	Cardiovascular events, mortality, admission due to fall/fracture, or admission with dementia/delirium	Positive correlation	Cohort study
	Clarkson *et al* [47]	Falls in older adults	Positive correlation	Cohort study
	Bishara *et al* [11]	Mortality, hospital admission and cognitive decline in people with dementia	Positive correlation	Cohort study
**AIS** [12]	Internal study	Peripheral ACH SEs	Positive correlation	Cross sectional study
	Vidal, Nathan *et al* [46]	Cognitive impairment in Schizophrenia	Positive correlation	Cross sectional study
	Lisibach *et al* [23]	In hospital mortality and length of stay	Positive correlation	Cohort study
	Hanlon *et al* [32]	Cardiovascular events, mortality, admission due to fall/fracture, or admission with dementia/delirium	Positive correlation	Cohort study
	Vidal *et al* [26]	Peripheral ACH SEs in BD	Positive correlation	Cohort study
**MARANTE** 13	Geßele *et al* [48]	Postoperative delirium	Positive correlation	Cohort study
	Vidal *et al* [26]	Peripheral ACH Ses in BD	Positive correlation	Cohort study
	Dinh *et al* [31]	Ability to predict falls	Limited value	RTC
**ABS** [14]	Vidal, Nathan *et al* [46]	Cognitive impairment in Schizophrenia	Positive correlation	Cross sectional study
	Clarkson *et al* [47]	Falls in older adults	Positive correlation	Cohort study
	Lisibach *et al* [23]	In hospital mortality and length of stay	Positive correlation	Cohort study
	Dinh *et al* [49]	Ability to predict falls	Limited value	RTC
	Vidal *et al* [26]	Peripheral ACH Ses in BD	Positive correlation	Cohort study

Scale	Clinical Outcome study	Outcome Evaluated against	Outcome of Evaluation	Type of study
**KABS** [15]	Vidal, Nathan *et al* [46]	Cognitive impairment in Schizophrenia	Positive correlation	Cross sectional study
	Lisibach *et al* [23]	In hospital mortality and length of stay	Positive correlation	Cohort study
	Vidal *et al* [26]	Peripheral ACH Ses in BD	Positive correlation	Cohort study
**BAAS** [16]	Lisibach *et al* [23]	In hospital mortality and length of stay	Positive correlation	Cohort study
	Vidal *et al* [26]	Peripheral ACH Ses in BD	Positive correlation	Cohort study
	De Lima *et al* [50]	Reduction in health-related quality of life in patients with multiple myeloma	Positive correlation	Cross sectional study
**CALS** [17]	Internal study	Cognitive impairment	Positive correlation	Cohort study
	Muglia *et al* [36]	Dysphagia in hospitalised patients	Positive correlation	Cohort study
	Vidal, Nathan *et al* [46]	Cognitive impairment in Schizophrenia	Positive correlation	Cross sectional study
	Mcquaid *et al* [37]			

References

1. Summers WK. A CLINICAL METHOD OF ESTIMATING RISK OF DRUG INDUCED DELIRIUM. 1978

2. Ancelin ML, Artero S, Portet F, Dupuy A, Touchon J, Ritchie K. Non-degenerative mild cognitive impairment in elderly people and use of anticholinergic drugs: longitudinal cohort study. *BMJ.* 2006;332(7539):455. doi:10.1136/bmj.38740.439664.de

3. Carnahan RM, Lund BC, Perry PJ, Pollock BG, Culp KR. The Anticholinergic Drug Scale as a Measure of Drug-Related Anticholinergic Burden: Associations With Serum Anticholinergic Activity. *The Journal of Clinical Pharma.* 2006;46(12):1481. doi:10.1177/0091270006292126

4. Rudolph JL, Salow MJ, Angelini MC, McGlinchey RE. The Anticholinergic Risk Scale and Anticholinergic
Adverse Effects in Older Persons. *ARCH INTERN MED.* 2008;168(5). doi:10.1001/archinternmed.2007.106

5. Boustani M, Campbell N, Munger S, Maidment I, Fox C. Impact of Anticholinergics on the Aging Brain: A Review and Practical Application. *Aging Health.* 2008;4(3):311. doi:10.2217/1745509x.4.3.311

6. Chew ML, Mulsant BH, Pollock BG, et al. Anticholinergic Activity of 107 Medications Commonly Used by Older Adults. *J American Geriatrics Society.* 2008;56(7). doi:10.1111/j.1532-5415.2008.01737.x

7. Ehrt U, Broich K, Larsen JP, Ballard C, Aarsland D. Use of drugs with anticholinergic effect and impact on cognition in Parkinson's disease: a cohort study. *Journal of Neurology, Neurosurgery & Psychiatry.* 2009;81(2):160. doi:10.1136/jnnp.2009.186239

8. Sittironnarit G, Ames D, Bush AI, et al. Effects of Anticholinergic Drugs on Cognitive Function in Older Australians: Results from the AIBL Study. *Dement Geriatr Cogn Disord.* 2025;31(3):173. doi:10.1159/000325171

9. Kalisch Ellett LM, Pratt NL, Ramsay EN, Barratt JD, Roughead EE. Multiple Anticholinergic Medication Use and Risk of Hospital Admission for Confusion or Dementia. *J American Geriatrics Society.* 2014;62(10):1916. doi:10.1111/jgs.13054

10. Sumukadas D, Mcmurdo MET, Mangoni AA, Guthrie B. Temporal trends in anticholinergic medication prescription in older people: repeated cross-sectional analysis of population prescribing data. *Age and Ageing.* 2013;43(4):515. doi:10.1093/ageing/aft199

11. Bishara D, Perera G, Harwood D, et al. The anticholinergic effect on cognition (AEC) scale—Associations with mortality, hospitalisation and cognitive decline following dementia diagnosis. *Int J Geriat Psychiatry.* 2020;35(9):1069. doi:10.1002/gps.5330

12. Briet J, Javelot H, Heitzmann E, et al. The anticholinergic impregnation scale: Towards the elaboration of a scale adapted to prescriptions in French psychiatric settings. *Therapies.* 2017;72(4):427. doi:10.1016/j.therap.2016.12.010

13. Klamer TT, Wauters M, Azermai M, et al. A Novel Scale Linking Potency and Dosage to Estimate Anticholinergic Exposure in Older Adults: the Muscarinic Acetylcholinergic

Receptor ANTagonist Exposure Scale. *Basic Clin Pharma Tox.* 2017;120(6):582. doi:10.1111/bcpt.12699

14. Kiesel EK, Hopf YM, Drey M. An anticholinergic burden score for German prescribers: score development. *BMC Geriatr.* 2018;18(1). doi:10.1186/s12877-018-0929-6

15. Jun K, Hwang S, Ah Y, Suh Y, Lee J. Development of an Anticholinergic Burden Scale specific for Korean older adults. *Geriatrics Gerontology Int.* 2019;19(7):628. doi:10.1111/ggi.13680

16. Nery RT, Reis AMM. Development of a Brazilian anticholinergic activity drug scale. *Einstein.* 2019;17(2):eAO4435.

17. Ramos H, Moreno L, Pérez-Tur J, Cháfer-Pericás C, García-Lluch G, Pardo J. CRIDECO Anticholinergic Load Scale: An Updated Anticholinergic Burden Scale. Comparison with the ACB Scale in Spanish Individuals with Subjective Memory Complaints. *JPM.* 2022;12(2). doi:10.3390/jpm12020207

18. Rube T., Ecorcheville A., Londos E., Modig S., Johansson P. Development of the Swedish anticholinergic burden scale (Swe-ABS). *BMC geriatrics.* 2023;23(1):518.

19. Kehman C.M.B., Schlunsen M., Kjeldsen LJ. Categorisation of Patients' Anticholinergic Burden at Admission and Discharge from the Geriatric Ward of Sonderjylland Hospital. *Pharmacy.* 2024;12(6) (pagination):Article Number: 160. Date of Publication: 01 Dec 2024.

20. Yamada S, Mochizuki M, Chimoto J, Futokoro R, Kagota S, Shinozuka K. Development of a pharmacological evidence-based anticholinergic burden scale for medications commonly used in older adults. *Geriatrics Gerontology Int.* 2023;23(7):558. doi:10.1111/ggi.14619

21. Mizokami F., Mizuno T., Taguchi R., et al. Development of the Japanese Anticholinergic Risk Scale: English translation of the Japanese article. *Geriatrics and Gerontology International.* 2024(pagination):Date of Publication: 2024.

22. Han L, Mccusker J, Cole M, Abrahamowicz M. Use of Medications With Anticholinergic Effect Predicts Clinical Severity of Delirium Symptoms in Older Medical Inpatients. *Arch Intern Med.* 2001:1099–1105. doi:10.1001/archinte.161.8.1099

23. Lisibach A, Gallucci G, Beeler PE, Csajka C, Lutters M. High anticholinergic burden at admission associated with in-hospital mortality in older patients: A comparison of 19 different anticholinergic burden scales. *Basic Clin Pharma Tox.* 2021;130(2):288. doi:10.1111/bcpt.13692

24. Tiisanoja A, Syrjälä A-H, Kullaa A, Ylöstalo P. Anticholinergic Burden and Dry Mouth in Middle-Aged People. *JDR Clinical & Translational Research.* 2019;5(1):62. doi:10.1177/2380084419844511

25. Salahudeen MS, Hilmer SN, Nishtala PS. Comparison of Anticholinergic Risk Scales and Associations with Adverse Health Outcomes in Older People. *J American Geriatrics Society.* 2015;63(1):85. doi:10.1111/jgs.13206

26. Vidal N, Brunet-Gouet E, Frileux S, et al. Comparative analysis of anticholinergic burden scales to explain iatrogenic cognitive impairment and self-reported side effects

in the euthymic phase of bipolar disorders: Results from the FACE-BD cohort. *European Neuropsychopharmacology.* 2023;77:67. doi:10.1016/j.euroneuro.2023.08.502

27. Conti MSB, Sañudo A, Ramos LR. Anticholinergic scales and their relation to polypharmacy, cognition, and functional losses in aged in Brazil. *Cien Saude Colet.* 2023;30(2). doi:10.1590/1413-81232025302.09332023.

28. Michail A, Almirza M, Alwaely F, Arany S. Anticholinergic burden of medications is associated with dry mouth and reflected in minor labial gland secretion. *Archives of Oral Biology.* 2023;156. doi:10.1016/j.archoralbio.2023.105824

29. Aldebert G, Faillie JL, Hillaire-Buys D, et al. Association of Anticholinergic Drug Use With Risk for Late Age-Related Macular Degeneration. *JAMA Ophthalmol.* 2018;136(7). doi:10.1001/jamaophthalmol.2018.1719

30. Andre L, Gallini A, Montastruc F, et al. Anticholinergic exposure and cognitive decline in older adults: effect of anticholinergic exposure definitions in a 3-year analysis of the multidomain Alzheimer preventive trial (MAPT) study. *Brit J Clinical Pharma.* 2018;85(1):71. doi:10.1111/bcp.13734

31. Dinh TS, Meid AD, Rudolf H, et al. Anticholinergic burden measures, symptoms, and fall-associated risk in older adults with polypharmacy: Development and validation of a prognostic model. *PLoS ONE.* 2023;18(1). doi:10.1371/journal.pone.0280907

32. Hanlon P, Quinn TJ, Gallacher KI, et al. Assessing Risks of Polypharmacy Involving Medications With Anticholinergic Properties. *Ann Fam Med.* 2020;18(2):148. doi:10.1370/afm.2501

33. Kersten H, Molden E, Tolo IK, Skovlund E, Engedal K, Wyller TB. Cognitive effects of reducing anticholinergic drug burden in a frail elderly population: a randomized controlled trial. *J Gerontol A Biol Sci Med Sci.* 2013;68(3):271–8. doi:10.1093/gerona/gls176

34. Hsu W, Huang S, Lu W, Wen Y, Chen L, Hsiao F. Impact of Multiple Prescriptions With Anticholinergic Properties on Adverse Clinical Outcomes in the Elderly: A Longitudinal Cohort Study in Taiwan. *Clin Pharma and Therapeutics.* 2021;110(4):966. doi:10.1002/cpt.2217

35. Crispo JAG, Willis AW, Thibault DP, et al. Associations between Anticholinergic Burden and Adverse Health Outcomes in Parkinson Disease. *PLoS ONE.* 2016;11(3). doi:10.1371/journal.pone.0150621

36. Muglia L, Beccacece A, Soraci L, et al. Anticholinergic drug exposure is associated with prevalence, worsening and incidence of dysphagia among hospitalized older adults. *The Journal of nutrition, health and aging.* 2025;29(5). doi:10.1016/j.jnha.2025.100507

37. Mcquaid GA, Duane SC, Ahmed N, Lee NR, Charlton R, Wallace GL. Increased anticholinergic medication use in middle-aged and older autistic adults and its associations with self-reported memory difficulties and cognitive decline. *Autism Research.* 2023;17(4):852. doi:10.1002/aur.3076

38. Ablett AD, Wood AD, Barr R, et al. A high anticholinergic burden is associated with a history of falls in the previous year in middle-aged women: findings from the Aberdeen

Prospective Osteoporosis Screening Study. *Annals of epidemiology.* 2018;28(8). doi:https://doi.org/10.1016/j.annepidem.2018.05.011

39. Tsai T, Loh C, Huang H. Anticholinergic medicines linked to cardiovascular events in older adults. *BMJ.* 2023. doi:10.1136/bmj.p2133

40. Stenbäck J, Tiisanoja A, Syrjälä A, Komulainen K, Hartikainen S, Ylöstalo P. High anticholinergic burden and hyposalivation and xerostomia in the elderly. *Acta Odontologica Scandinavica.* 2023:1. doi:10.1080/00016357.2023.2166105

41. Ziad A, Olekhnovitch R, Ruiz F, et al. Anticholinergic drug use and cognitive performances in middle age: findings from the CONSTANCES cohort. *J Neurol Neurosurg Psychiatry.* 2018;89(10):1107. doi:10.1136/jnnp-2018-318190

42. Huang W, Yang AS, Tsai DH, Shao S, Lin S, Lai EC. Association between recently raised anticholinergic burden and risk of acute cardiovascular events: nationwide case-case-time-control study. *BMJ.* 2023. doi:10.1136/bmj-2023-076045

43. Tiisanoja A, Syrjälä A-H, Kullaa A, Ylöstalo P. Anticholinergic Burden and Dry Mouth in Middle-Aged People. *JDR Clinical & Translational Research.* 2019;5(1):62. doi:10.1177/2380084419844511

44. Hauff J, Rottenkolber M, Oehler P, et al. Single and combined use of fall-risk-increasing drugs and fracture risk: a population-based case–control study. *Age and Ageing.* 2023;52(6). doi:10.1093/ageing/afad079

45. Clarke CL, Sniehotta FF, Vadiveloo T, Donnan PT, Witham MD. Association Between Obj ectively Measured Physical Activity and Opioid, Hypnotic, or Anticholinergic

Medication Use in Older People: Data from the Physical Activity Cohort Scotland Study. *Drugs Aging.* 2018;35(9):835. doi:10.1007/s40266-018-0578-7

46. Vidal N, Roux P, Urbach M, et al. Comparative analysis of anticholinergic burden scales to explain iatrogenic cognitive impairment in schizophrenia: results from the multicenter FACE-SZ cohort. *Front Pharmacol.* 2024;15. doi:10.3389/fphar.2024.1403093

47. Clarkson L, Griffiths A, Ng S, Lam AK, Khoo TK. Preadmission medications and recent falls in older inpatients: an observational study. *Int J Clin Pharm.* 2025. doi:10.1007/s11096-024-01859-y

48. Geßele C, Rémi C, Smolka V, et al. Anticholinergic Exposure, Drug Dose and Postoperative Delirium: Comparison of Dose-Related and Non-Dose-Related Anticholinergic Burden Scores in a Retrospective Cohort Study of Older Orthopaedic and Trauma Surgery Patients. *Drugs Aging.* 2024;41(12):1003. doi:10.1007/s40266-024-01159-0

49. Dinh T.S., Meid A.D., Rudolf H., et al. Anticholinergic burden measures, symptoms, and fall-associated risk in older adults with polypharmacy: Development and validation of a prognostic model. *PLoS ONE.* 2023;18(1 January) (pagination):Article Number: e0280907. Date of Publication: 01 Jan 2023.

50. De Lima MSR, De Pádua CAM, De Miranda Drummond PL, et al. Health-related quality of life and use of medication with anticholinergic activity in patients with multiple myeloma. *Support Care Cancer.* 2023;31(7). doi:10.1007/s00520-023-07835-y
